# Trade off-free entanglement stabilization in a superconducting qutrit-qubit system

**DOI:** 10.1038/s41467-022-31638-0

**Published:** 2022-07-09

**Authors:** T. Brown, E. Doucet, D. Ristè, G. Ribeill, K. Cicak, J. Aumentado, R. Simmonds, L. Govia, A. Kamal, L. Ranzani

**Affiliations:** 1 Department of Physics and Applied Physics, University of Massachusetts, Lowell, MA 01854 USA; 2 Quantum Engineering and Computing, Raytheon BBN, Cambridge, MA 02138 USA; 3 National Institute of Standards and Technology, 325 Broadway, Boulder, CO 80305 USA; 4 Present Address: Keysight Technologies, Cambridge, MA 02139 USA

**Keywords:** Quantum information, Qubits

## Abstract

Quantum reservoir engineering is a powerful framework for autonomous quantum state preparation and error correction. However, traditional approaches to reservoir engineering are hindered by unavoidable coherent leakage out of the target state, which imposes an inherent trade off between achievable steady-state state fidelity and stabilization rate. In this work we demonstrate a protocol that achieves trade off-free Bell state stabilization in a qutrit-qubit system realized on a circuit-QED platform. We accomplish this by creating a purely dissipative channel for population transfer into the target state, mediated by strong parametric interactions coupling the second-excited state of a superconducting transmon and the engineered bath resonator. Our scheme achieves a state preparation fidelity of 84% with a stabilization time constant of 339 ns, leading to a 54 ns error-time product in a solid-state quantum information platform.

## Abstract (web summary)

The approach to stabilizing a quantum state by coupling to engineered reservoirs is limited by a trade-off between state fidelity and stabilization rate. Here the authors implement a protocol based on parametric system-bath coupling to achieve fast and high-fidelity Bell state stabilization in a qutrit-qubit system.

## Introduction

Entanglement is a fundamental property of quantum systems and is essential to achieve quantum advantage in almost any application of quantum information processing, such as sensing^1^, communication^2^ and computing^3^. Typically, entanglement is created by applying a sequence of single and two-qubit unitaries; however the resulting states are subject to decoherence caused by coupling to the surrounding environment^4^. In the absence of active error correction^5–10^, decoherence limits the circuit depth and, consequently, the size of the entangled state that can be produced. Moreover, this approach is sensitive to state preparation and measurement errors which accumulate as the complexity and size of the quantum system increases. An attractive alternative for quantum state preparation is quantum reservoir engineering, where a quantum system is steered to a desired entangled state by coupling it to an auxiliary system (“engineered reservoir”) that induces strong, non-local dissipation on the target system^11–21^. In addition to being immune to initialization errors, the target state remains stabilized for times much longer than the coherence time of the individual qubits, ensuring the entangled state is always available on demand.

Though there have been several demonstrations of dissipative stabilization in diverse quantum information platforms, such as superconducting qubits^14–16,22–25^, trapped ions^17,26–29^, atomic systems^30^, and NV centers^31^, almost all reported schemes have been hindered by unavoidable coherent leakage out of the target state that cannot be suppressed without also reducing the repumping rate into the desired state. This issue leads to a trade off in reservoir engineering: the product of minimum steady-state error (*ε*_*∞*_) and stabilization time (*τ*) is a constant that is independent of the engineered dissipation rate, implying that perfect entanglement stabilization cannot be achieved at rates faster than the uncontrolled dissipation rates^32^. This severely limits the prospects of reservoir engineering both in terms of (1) usability with regard to implementation in systems with strong local (uncontrolled) decoherence, which ironically stand to gain most from such stabilization techniques, and (2) scalability with regard to state preparation in large quantum networks, where it becomes increasingly harder for the stabilization rate to beat the cumulative local decoherence, which scales (at least) linearly with system size^33–35^.

Nonetheless, as shown by our recent work^11^ such a trade off is not a fundamental limitation of autonomous state stabilization, but is a consequence of driven-dissipative schemes that transfer population into a target state at a rate limited by a drive strength which needs to remains weak (or “perturbative”) as compared to the dressed linewidth to maintain resonant pumping. As a result, in the strong coupling regime the desired entangled state ceases to be the dark state of the dynamics (i.e., an eigenstate of the drive Hamiltonian that is simultaneously also a null state of the engineered dissipation). The concurrent scaling of terminal fidelity and stabilization rate can be achieved instead by engineering entanglement stabilization protocols that do not cause increasing coherent leakage under strong coupling, so that fast stabilization can be achieved without sacrificing fidelity to a fixed entangled state. Such protocols are limited only by incoherent error sources that are much slower than the stabilization rate.

In this work, we implement a trade off-free Bell-state stabilization protocol in a superconducting circuit-QED system comprising two transmons parametrically coupled to a common lossy resonator that acts as an engineered reservoir. We engineer a purely dissipative channel for population transfer into the target Bell state via parametric coupling to the third level of the transmon, and without any direct coherent coupling into or out of it, making it an eigenstate of the drive Hamiltonian and also a dark state of the engineered dissipation. Our scheme attains a steady-state fidelity of 84% with a time-constant of 339 ns achieving the error-time product *ε*_*∞*_*τ* ≃ 54 ns. Furthermore we verify that the steady state error and preparation time are linearly correlated. Notably, the reported protocol is the minimal instance of trade off-free stabilization physics that employs only continuous unconditional driving and linear (engineered) dissipation.

## Results

**Fig. 1:**
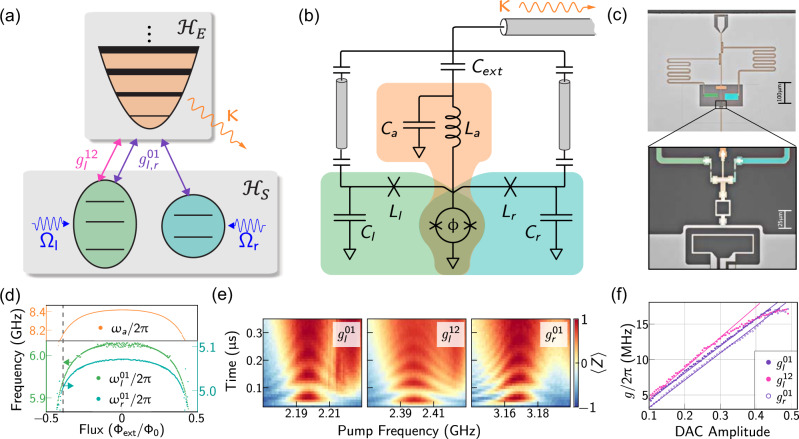
Device design and characterization. **a** Schematic diagram showing a lossy resonator with linewidth *κ* coupled to a qutrit-qubit system via three parametric drives. Rabi drives resonant with the 0–1 transition are also applied to both the qutrit and the qubit. **b** Circuit realization of the scheme in **a** showing two superconducting transmons (green and turquoise) and central resonator (orange), as well as the dedicated readout resonators (gray). The transmons and central resonator share a SQUID that implements parametric couplings (${g}_{k}^{n,n+1}$). **c** Optical micrographs of the device layout (left) and a magnified view of the junctions and the SQUID coupler (right). The external bias line used to pump the SQUID can be seen at the bottom of the device. The resonator consists of an array of 10 Josephson junctions, each having a critical current *I*_*ac*_ ≈ 540 nA, in series with a fixed capacitor. **d** Measured 0–1 transition frequency for each transmon and resonator center frequency as a function of flux through the SQUID loop. The operating flux bias is indicated with a dashed-black line. **e** Time-domain parametric swaps measured as a function of pump frequency for each of the three parametric drives in Eq. (1). From left to right, the transmons are initialized in $\left|10\right\rangle$, $\left|20\right\rangle$, and $\left|01\right\rangle$ respectively. **f** Parametric coupling rates measured as a function of pump amplitude. Solid lines are linear fits to the data; the nonlinear response of ${g}_{l}^{12}$ at higher drive amplitude is due to enhanced mixer saturation at the corresponding IF frequency of 150 MHz, as compared to the other drives, which use 50 MHz.

Figure 1a depicts the general scheme, in which a qutrit-qubit system is coupled to a lossy resonator using bilinear parametric interactions. Our circuit-QED implementation in Fig. 1b consists of two transmon qubits coupled to a superconducting resonator. To reduce the circuit size, we implemented the superconducting resonator as a capacitor in series with a chain of Josephson junctions, each having a critical current of 540 nA. The parametric interactions are realized by grounding the resonator and the transmon junctions through a shared superconducting quantum interference device (SQUID) loop, which acts as a flux-tunable inductor. Through sinusoidal modulation of the flux through the SQUID loop, ${{\Phi }}(t)={{{\Phi }}}_{{{{{{{{\rm{ext}}}}}}}}}+{\sum }_{j}{{{\Phi }}}_{j}\cos ({\omega }_{j}t+{\phi }_{j})$, pairwise couplings can be activated between any pair of elements via the choice of the pump frequency *ω*_*j*_. We show the layout of the experimental device in Fig. 1c, with detailed parameters listed in Supplementary Table 1.

We simultaneously activate the parametric couplings depicted in Fig. 1a by flux-pumping the SQUID at the sideband frequencies ${\omega }_{a}\pm {\omega }_{k}^{n,n+1}$, corresponding to the desired transition frequencies of the transmon *k* = *l*, *r*. Specifically, we pump the two red-sideband frequencies ${\omega }_{a}-{\omega }_{l}^{01}$ and ${\omega }_{a}-{\omega }_{l}^{12}$ corresponding to the 0–1 and 1–2 transitions for transmon *l*, and the red-sideband at ${\omega }_{a}-{\omega }_{r}^{01}$ corresponding to the 0–1 transition of transmon *r*. In conjunction with Rabi drives on the 0–1 transitions of each qubit, this leads to an effective interaction Hamiltonian of the form (see Supplementary Note 1):

1 $${H}_{{{{{{{{\rm{I}}}}}}}}} =	\, {a}^{{{{\dagger}}} }\left(\frac{{g}_{l}^{12}}{2}{e}^{i{\phi }_{l}^{12}}{\left|1\right\rangle }_{l}\left\langle 2\right|+\mathop{\sum}\limits_{k\in \{l,r\}}\frac{{g}_{k}^{01}}{2}{e}^{i{\phi }_{k}^{01}}{\left|0\right\rangle }_{k}\left\langle 1\right|\right)\\ 	+\mathop{\sum}\limits_{k\in \{l,r\}}\frac{{{{\Omega }}}_{k}^{01}}{2}{e}^{i{\theta }_{k}}{\left|0\right\rangle }_{k}\left\langle 1\right|+h.c.,$$

where we have moved to a frame defined w.r.t. the free Hamiltonian and discarded off-resonant counter-rotating terms (see Supplementary Note 2). Here we approximate the resonator as a harmonic oscillator, since we estimate its anharmonicity to be *α* ≈ 80−100 kHz. The parametric couplings shuttle excitations between the transmon levels and the lossy resonator^14,36,37^, leading to an engineered quasi-local dissipator ${{{{{{{\mathcal{D}}}}}}}}\left[{L}_{{{{{{{{\rm{eff}}}}}}}}}\right]$ acting on the transmons with:

2 $${L}_{{{{{{{{\rm{eff}}}}}}}}}={c}_{l}^{12}{\left|1\right\rangle }_{l}\left\langle 2\right|+\mathop{\sum}\limits_{k\in \{l,r\}}{c}_{k}^{01}{\left|0\right\rangle }_{k}\left\langle 1\right|.$$

The coefficients ${c}_{k}^{n,n+1}$ are functions of the resonator decay rate *κ* and the corresponding parametric pump amplitudes (${g}_{k}^{n,n+1}$) and phases (${\phi }_{k}^{n,n+1}$). In particular, the phases ${\phi }_{k}^{n,n+1}$ are needed to perform coherent control of the stabilized state, as explained later. We choose our coupling rates so that the target Bell state $\left|\psi \right\rangle$ is an eigenstate of the Hamiltonian in Eq. (1) and satisfies ${L}_{{{{{{{{\rm{eff}}}}}}}}}\left|\psi \right\rangle =0$^11,38^. For example, in order to prepare the state $\left|\psi \right\rangle =(1/\sqrt{2})(\left|01\right\rangle +{e}^{i\phi }\left|10\right\rangle )$ we set ${g}_{l}^{01}=-{e}^{i\phi }{g}_{r}^{01}$ and ${{{\Omega }}}_{l}^{01}=-{e}^{i\phi }{{{\Omega }}}_{r}^{01}$.

We emphasize that this qutrit-qubit scheme is the minimal system to realize trade off-free stabilization of a two-qubit maximally entangled state, using only unconditional continuous-wave driving and linear dissipation. Specifically, restricting to linear engineered dissipation allows using bilinear interactions, which are easy to implement via three-wave parametric mixing in Josephson circuits. Further, leveraging the tunability of parametric interactions with pump amplitude, coupling strengths can be tuned in situ from weak to strong coupling to find the optimal drives required to achieve lowest error. In our experiment, we bias the SQUID coupler at Φ_ext_ = −0.39Φ_0_ corresponding to transmon 0–1 transition frequencies of ${\omega }_{l}^{01}=2\pi \times 5.928\; {{{{{\rm{GHz}}}}}}$ and ${\omega }_{r}^{01}=2\pi \times 4.993\; {{{{{\rm{GHz}}}}}}$ and center frequency of the resonator *ω*_*a*_ = 2*π* × 8.124 GHz (Fig. 1d). The noise in spectroscopy visible at zero flux in Fig. 1d is due to the low readout fidelity caused by weak coupling of the readout cavity of transmon *l* to the input transmission line. We characterize the parametric drive amplitudes ${g}_{l,r}^{01}$ by initializing each transmon in state ${\left|1\right\rangle }_{l,r}$ and subsequently turning on the respective red-sideband ${\omega }_{a}-{\omega }_{l,r}^{01}$ to measure the coherent swaps with the resonator, see Fig. 1e, f. Similarly, we measure ${g}_{l}^{12}$ by initializing the left transmon in the state ${\left|2\right\rangle }_{l}$ using a sequence of *π*^01^, *π*^12^ pulses, the first resonant with the ${\omega }_{l}^{01}$ and the second resonant with the ${\omega }_{l}^{12}={\omega }_{l}^{01}+{\alpha }_{l}$, where the anharmonicity *α*_*l*_ = −2*π* × 198 MHz (Supplementary Note 4). We then measure the coherent swap between the states ${\left|2\right\rangle }_{l}{\left|0\right\rangle }_{a}$ and ${\left|1\right\rangle }_{l}{\left|1\right\rangle }_{a}$ under the action of red-sideband at ${\omega }_{a}-{\omega }_{l}^{12}$. In Fig. 1e we show an example of time-domain oscillations, indicating coherent swap with decay time approximately equal to 2/*κ*, which is consistent with the hybridization between the transmon transitions and the lossy resonator. We fit the oscillations on resonance corresponding to each transition to a decaying sinusoid and extract the parametric coupling rate from the swap period 1/*g*; we do this for different drive amplitudes and measure coupling rates up to *g* = 2*π* × 17.5 MHz (Fig. 1f).

**Fig. 2:**
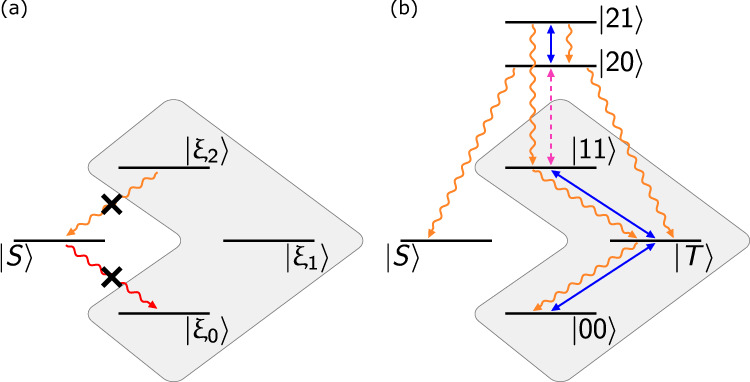
Minimality of proposed scheme. **a** Quasi-local dissipation engineered with red- and blue-sideband qubit-oscillator interactions: it is not possible to protect the target Bell state $\left|S\right\rangle$ from decay (red arrow) without also suppressing any repumping channel from the orthogonal subspace into the target state (orange arrow). **b** The effective action of our protocol on the reduced qutrit-qubit subspace. Note how the qutrit levels provide the triplet subspace an indirect path to decay into the singlet subspace (Supplementary Fig. 1).

The minimality of the scheme, i.e., why it is sufficient to include one extra level to achieve trade off-free stabilization, can be seen using the following simple argument. Let us assume, without loss of generality, that we want to stabilize the singlet state $\left|S\right\rangle =(1/\sqrt{2})(\left|01\right\rangle -\left|10\right\rangle )$. The most general jump operator restricted to a two-qubit (four-level) space that satisfies $L\left|S\right\rangle =0$ is *L* = *c*_−_*J*^−^ + *c*_+_*J*^+^ where *J*^±^ are the total spin raising and lowering operators. Similarly, if $\left|S\right\rangle$ is an eigenstate of the system Hamiltonian, the latter commutes with the total spin $\left[H,{J}^{2}\right]=0$. We conclude that since every generator commutes with *J*^2^, the total spin is a conserved quantity and hence the qubits cannot be stabilized into $\left|S\right\rangle$ (corresponding to *J* = 0) when initialized in a state corresponding to different total spin (*J* = 1). Figure 2a illustrates the problem—that in the process of engineering $\left|S\right\rangle$ as the steady state of dissipation, we completely decouple it from the rest of the Hilbert space. The previous argument can be extended to the stabilization of any Bell state, by redefining the spin operators up to a local unitary on either qubit.

In the past^38^, this issue has been bypassed by stabilizing an “approximate” Bell state, $\left|\psi \right\rangle ={{{{{{{{\mathcal{N}}}}}}}}}_{\delta }(\left|S\right\rangle +\delta \left|\xi \right\rangle )$ with ${L}^{{{{\dagger}}} }\left|\xi \right\rangle \ne 0$, thus allowing the use of interactions which do not conserve the total spin. This approach, however, only enables a perturbative stabilization of Bell states, which results in a trade off between intrinsic error of the protocol and stabilization rate. In contrast, the procedure presented here eliminates this problem by expanding the system Hilbert space minimally to a qutrit-qubit system and realizing a scheme with ideally no intrinsic error (see Supplementary Note 2 and Supplementary Fig. 1). The sideband coupling to the second-excited state of the qutrit (${g}_{l}^{12}$) leads to a jump operator as indicated in Eq. (2) that does not preserve the “total spin” on the qubit-qubit subspace but still supports $\left|S\right\rangle$ as a steady state. Figure 2b illustrates the mechanism of our protocol after adiabatic elimination of the oscillator^39^. There is no direct coupling between the singlet and triplet subspaces, either dissipative or coherent. Instead, the additional levels included in the Hilbert space due to the second-excited state of the qutrit mediate a pathway for population to decay from the triplet to the singlet subspace in a multi-step process.

We note that extending the Hilbert space is not the only method available to mitigate the trade off between error and time of stabilization. Previous works have considered time-dependent control of drives^40^ or conditional (number-selective) driving^15^ as a way to increase stabilization speed. Nonetheless, the interactions required are either more complex to control, and/or rely on a combination of both static and parametric interactions. More crucially, such protocols are susceptible to coherent leakage for drive amplitudes comparable to dispersive shifts (few MHz), which reimposes the error-time trade off^11^. On the other hand, with higher level driving the frequency of the nearest counter-rotating terms is determined by the anharmonicity of the atom (few 100 MHz for transmons), allowing usage of larger drive amplitudes and, consequently, stronger engineered dissipation rates (see Supplementary Fig. 2).

### Stabilization mechanism and performance

**Fig. 3:**
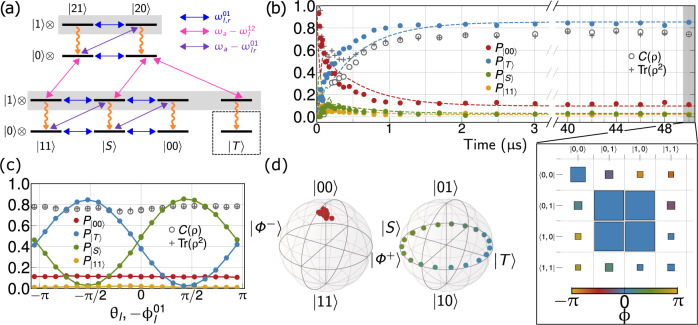
Stabilization protocol and results. **a** Energy level diagram showing the action of Ω_*l*,*r*_ (blue), ${g}_{l,r}^{01}$ (purple), ${g}_{l,r}^{12}$ (magenta), and *κ* (orange). Only the first two energy levels of the resonators are shown for clarity. **b** Stabilization trajectory for transmons initialized in $\left|00\right\rangle$ obtained for Ω_*l*,*r*_ = 2*π* × 7.2 MHz, ${g}_{l,r}^{01}=2\pi \times 7.5$ MHz, ${g}_{l}^{12}=2\pi \times 13.1$ MHz, *κ* = 2*π* × 4.73 MHz, showing target state (here $\left|T\right\rangle$) fidelity of 84% fidelity stabilized for 50 μs. The dashed line corresponds to trajectories obtained using master equation simulations. The density matrix at *t* = 50 μs, reconstructed using quantum state tomography, shows that most of steady-state error is accounted for by decay into $\left|00\right\rangle$. The size of the squares in the tomogram represent the magnitude, while the color represents the phase of the density matrix element. **c** Coherent control of the stabilized state, with simultaneous tuning of the phases of Rabi (Ω_*l*_) and parametric drives (${g}_{l}^{01}$), maintaining ${\phi }_{l}^{01}+{\theta }_{l}=0$. The populations are shown at a fixed time *t* = 2 μs as a function of drive phase and remaining drive phases fixed. The average purity and concurrence over the 2*π* rotation are 77%. **d** Two-qubit Bloch sphere representations for (normalized) projections of steady state in odd- and even-parity manifolds.

The stabilization procedure implemented by the Hamiltonian in Eq. (1), in conjunction with the resonator decay (*κ*), is able to stabilize any odd-parity Bell state through appropriate choice of phases *ϕ*_*k*_, *θ*_*k*_. Figure 3a shows the full energy level diagram, including the resonator levels, depicting the mechanism for stabilization of triplet state $\left|T\right\rangle =(1/\sqrt{2})(\left|01\right\rangle +\left|10\right\rangle )$ as an example. Here, the two 0–1 sideband drives are set out-of-phase (purple arrows) to selectively couple only the orthogonal state $\left|S\right\rangle$ to the even-parity states in the one-excitation manifold of the resonator. The out-of-phase Rabi drives (blue arrows) couple $\left|00\right\rangle$ (and $\left|11\right\rangle$) to $\left|S\right\rangle$ and prevent the system from being trapped in $\left|00\right\rangle {\left|0\right\rangle }_{a}$. Finally, the 1–2 sideband drive (magenta arrows) couples the second-excited state of the left transmon to the states in the one- and two-excitation manifolds of the resonator. The combined action of the three parametric and two direct drives pumps the population into $\left|T\right\rangle {\left|1\right\rangle }_{a}$, which then quickly decays to the target state $\left|T\right\rangle {\left|0\right\rangle }_{a}$. Crucially, no drive acts on $\left|T\right\rangle {\left|0\right\rangle }_{a}$ directly, suppressing any coherent leakage out of the target state as explained before.

For demonstrating stabilization, we initialize both transmons in their ground state since it requires no prior active preparation, simultaneously turn on all three parametric drives and two Rabi drives for a fixed time *t*, and finally perform two-qubit quantum state tomography to reconstruct the evolution of the two-qubit state as a function of *t*. We note here that stabilized state is independent of the initial state of the qubits; data for different initial states are discussed in (Supplementary Fig. 7).

**Fig. 4:**
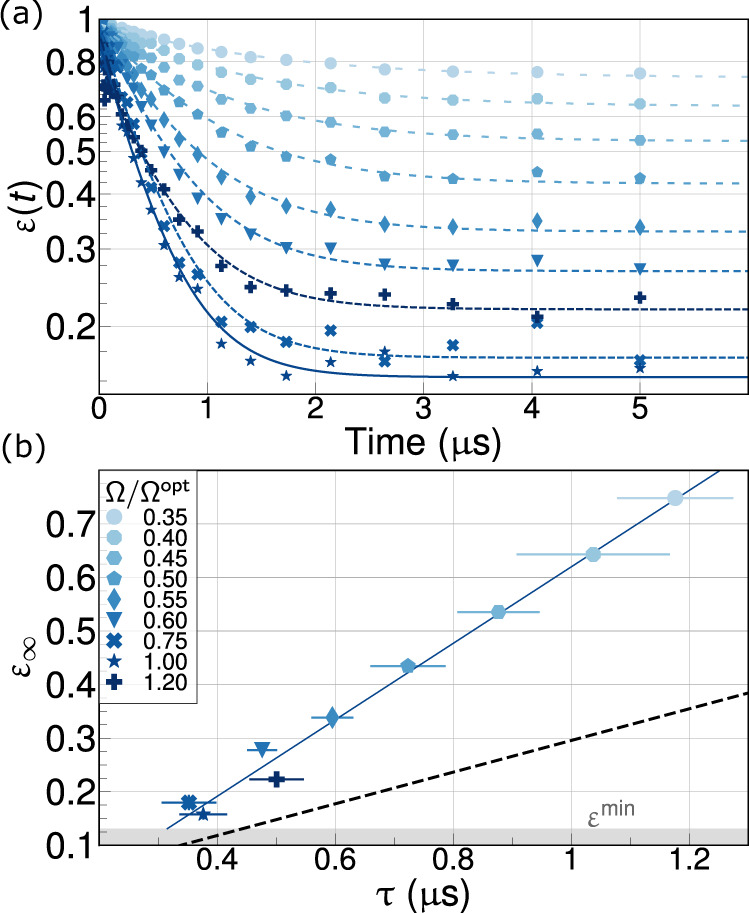
Trade off-free performance scaling. **a** Measurement of preparation error as a function of time, *ε*(*t*), for different Rabi drive amplitudes of ${{{\Omega }}}_{l,r}^{01}$. **b** Parametric plot of the steady-state error *ε*_*∞*_ and the stabilization time constant *τ*, obtained from fitting the data in (**a**) showing the expected linear scaling. The minimum *ε*_*∞*_ and *τ* are measured at ${{{\Omega }}}_{l,r}^{{{{{{{{\rm{opt}}}}}}}}}\approx 1.5\kappa$. The minimum error ${\varepsilon }_{\infty }^{\min }$ is close to theoretically achievable value for experimental *κ* (shown as the gray floor), (Supplementary Fig. 11). The dotted line is the error-time scaling obtained from a simulation of Eq. (1) and has slope equal to 1/*T*_*B*_ with *T*_*B*_ = 3.38 μs, independent of drive amplitudes, in accordance with the semi-classical estimate discussed in the “Methods” section. The deviation of experimentally observed slope from 1/*T*_*B*_ is quantitatively explained by including a residual drive detuning and imbalance in the sideband amplitude (Supplementary Fig. 12).

We examine the stabilization time constant, *τ*, and steady-state error, *ε*_*∞*_, from fitting the dynamical error for a given set of drive parameters as $\varepsilon (t)={\varepsilon }_{\infty }+\tilde{\varepsilon }\exp (-t/\tau )$, where $\varepsilon (t)=1-{{{{{{{\rm{Tr}}}}}}}}\{\rho (t){{\mathbb{I}}}_{{{{{{{{\rm{res}}}}}}}}}\otimes \left|T\right\rangle \left\langle T\right|\}$^11^. With the Rabi drives tuned to their optimal coupling strength, the average stabilization trajectory displays an exponential behavior with a characteristic 1/*e* time of 339 ns as it approaches its steady state fidelity of 84(1)%, found from the average and standard deviation of the data points between 10 and 50 μs (Fig. 3b). The remaining population resides primarily in the ground state $\left|00\right\rangle$ (11%), with small residuals in $\left|11\right\rangle$ (2%) and the orthogonal Bell state $\left|S\right\rangle$ (2%). We verified that the qubits remain in the steady state for as long as the pumps are on up to 50 μs, which is about 10× longer than the timescale set by the decoherence time of each qubit ${T}_{2}^{* }$ (Supplementary Table 1). In Fig. 3b we also show a full master equation simulation of the system and a tomogram of the reconstructed two-qubit density matrix at *t* = 50 μs. The simulations, which are entirely based on independently measured device parameters, predict the correct target state fidelity within our measurement uncertainty. The measured convergence time is about 15% faster than predicted by the theory for the measured drive amplitudes, which we attribute to residual drive detuning and imbalance, see also Fig. 4 and Supplementary Fig. 12.

We separately characterize leakage out of the qubit manifold by measuring the population of the ${\left|2\right\rangle }_{l}$ state, which peaks at *t* = 150 ns and then drops to less than 2.5% at *t* > 1 μs. In the Supplementary (Supplementary Note 5 and Supplementary Figs. 9 and 10) we provide details of obtaining a numerical bound on the error caused by this high-dimensional leakage (<1%) in the steady state reconstructed from two-qubit state tomography.

By exploiting the tunable nature of parametric interactions, we realize in situ coherent control within a fixed parity manifold. For example, the stabilized Bell state can be rotated by tuning the phases of the 0–1 sidebands and Rabi drives while ensuring ${\phi }_{l}^{01}+{\theta }_{l}={\phi }_{r}^{01}+{\theta }_{r}$. Such phase tuning allows for selection of any maximally entangled state while maintaining the purity, *P*(*ρ*), and concurrence, *C*(*ρ*), of the two-qubit state, as demonstrated in Fig. 3c. Additionally, it is possible to continuously move along the longitude from $\left|01\right\rangle$ to $\left|10\right\rangle$ by changing the ratio between ${g}_{l}^{01}/{g}_{r}^{01}$ and Ω_*l*_/Ω_*r*_. The post-selected two-qubit state (Fig. 3d) shows an average purity of 95% and 80% in odd- and even-parity manifolds, respectively. Using these numbers to model the full two-qubit state as a probabilistic mixture of even and odd parity subspaces, *ρ*_4×4_ = *xρ*_even_ ⊕ (1 − *x*)*ρ*_odd_ allows us to extract an improved post-selected fidelity of 97.25(5)% for the odd-parity Bell state averaged across the range of phases. This fidelity corresponds to the state obtained by performing an ideal parity measurement and projecting the stabilized state onto the odd-parity manifold^15^. In Fig. 3d we used the symbols ${{{\Phi }}}^{\pm }=(1/\sqrt{2})\left|00\right\rangle \pm \left|11\right\rangle$ for the even-parity maximally entangled states.

The main distinctive feature of our stabilization protocol is the concurrent scaling of preparation time and steady-state error. This is confirmed by the data presented in Fig. 4 which shows a linear relationship between *τ* and *ε*_*∞*_; both decrease as the Rabi drive strength is increased, reaching a minimum near the optimal ${{{\Omega }}}_{l,r}^{{{{{{{{\rm{opt}}}}}}}}}\approx 1.5\kappa$. Simulations of both the full and reduced system (the latter obtained via adiabatic elimination of the resonator) confirm the linear error-time scaling, with the slope determined almost entirely by the total decoherence rate of the stabilized Bell state $1/{T}_{B}={\sum }_{k\in l,r}? > ({\gamma }_{k}^{01}+{\gamma }_{k}^{11})/2$, i.e., *ε*_*∞*_/*τ* ≈ 1/*T*_*B*_. Here ${\gamma }_{k}^{01}$ is the 0–1 relaxation rate and ${\gamma }_{k}^{11}$ is the pure dephasing rate. The linear relationship between *ε*_*∞*_ and *τ* indicates that the steady-state error is due to competition between engineered dissipation and local decoherence rates rather than any coherent error process. This is further confirmed by the fact that the data match the theory more closely at high drive strengths, where the engineered dissipation rate is stronger.

The line corresponding to the experimentally measured stabilization error and time has a steeper slope than 1/*T*_*B*_ (c.f. Fig. 4b). Our simulations agree with the measured data when we include the effect of parametric crosstalk-induced drive detuning and amplitude imbalance, both of which lead to coherent leakage out of the target state^11^. The fitted detunings (about 400 kHz on average) are a small fraction (≤3%) of the measured power-dependent frequency shift of the right transmon (18 MHz), (see Supplementary Fig. 11 for details). We stress here that this coherent leakage remains weak compared to the engineered dissipation strength and, therefore, does not alter the expected error-time linear scaling.

To assess the performance reported here against previous experiments we compare the product of ${\varepsilon }_{\infty }^{\min }$ and *τ*, which can be thought of as an “inverse gain-bandwidth product” for state preparation. Note that in previous experiments, unlike our case, the product between *ε*_*∞*_ and *τ* is a constant. Nonetheless, we can still use this product as a composite figure of merit to compare performance across different protocols. Our experiment yields ${\varepsilon }_{\infty }^{\min }\tau =54{{{{{\rm{ns}}}}}}$, which is 5x - 6x lower than previous implementations of continuous-wave state stabilization in superconducting circuits (Table 1). We also propose an “information-theoretic” metric that allows to compute the upper bound on error-free output information generated by a multiple repetitions of a stabilization protocol. To this end, we model a stabilization cycle as a noisy binomial channel with the maximum success probability set by *p*(*t*) = 1 − *ε*(*t*) and the number of uses set by the ratio *n*_*c*_ = *T*_*c*_/*τ*, where ${T}_{c}^{-1}$ is the repetition rate of the experiment. Assuming the output to be a continuous normal distribution in the limit of large *n*_*c*_, this leads to the following expression for entanglement efficiency ${{{{{{{{\mathcal{E}}}}}}}}}_{e}$ for a given scheme:

3 $${{{{{{{{\mathcal{E}}}}}}}}}_{e}({T}_{c})=\frac{\tau }{{T}_{c}}\left(\mathop{\lim }\limits_{t\to {T}_{c}}{\log }_{2}\left[1+\frac{1-\varepsilon (t)}{\varepsilon (t)}\frac{{T}_{c}}{\tau }\right]\right).$$

This can be understood as the maximum “rate" at which the protocol can encode a continuous stream of bit-pairs into e-bits. In the derivation of Eq. (3) we assume that the noise of the channel is independent of the initial state (or the channel input) which is a reasonable approximation for steady states that are globally asymptotically stable, such as the one engineered by the protocol here. For long repetition times, steady state or minimum error, can be used to calculate the relevant efficiency. Last row of Table 1 quotes the upper bound on the average information (here number of e-bits) generation capacity, ${I}_{e}(x)=(x/\tau ){{{{{{{{\mathcal{E}}}}}}}}}_{e}(x)$, at a fixed time set by the decoherence rates of different platforms. As detailed in the supplement (Supplementary Note 6 and Supplementary Fig. 13), long stabilization times limit the capacity for short *T*_*c*_, leading to an overall low capacity for trapped-ion implementation reported in ref. ^17^ where ${T}_{2}^{* }\ll \tau$.

**Table 1:** Comparison of performance with previous implementations.

	Lin^17^	Liu^22^	K.S.^13^	This work
${T}_{2}^{* }$	100 μs	9 μs	2.6 μs	5.6 μs
		10 μs	3.0 μs	4.5 μs
${F}_{\infty }^{\max }$	77%	76%	71%	84%
*τ*	>1 ms	780 ns	760 ns	339 ns
${\varepsilon }_{\infty }^{\min }\tau$	>200 μs	187 ns	220 ns	54 ns
*I*_*e*_ ($\bar{{T}_{2}^{* }}$)	0.01	5.29	3.21	6.23

The quoted performance metrics are for continuous-wave (CW) driven and autonomous protocols, without any post-selection, similar to the one in the present work (see “Methods”). The composite metrics *ε*_∞_*τ* and *I*_*e*_(*x*) provide a platform-agnostic means for comparing different stabilization protocols.

## Discussion

In this work we have demonstrated an autonomous scheme which implements fast and high-fidelity Bell state stabilization in a qutrit-qubit system. Use of parametric system-bath interactions allows operating the protocol with strong drive strengths (“engineered” dissipation)—a regime which has hitherto remained inaccessible to reservoir engineering protocols based on resonant interactions. We verify that the preparation error scales linearly with the stabilization time constant, achieving a minimum error-time product of ${\varepsilon }_{\infty }^{\min }\tau =54$ ns for optimal drive strengths. The concurrent suppression of error-time product with drive amplitude results from a simultaneous minimization of drive-dominated and dissipation-dominated errors^11^, highlighting a crucial principle for design of reservoir engineering schemes. Further, we implemented continuous-wave coherent control and in situ target state selection leveraging the phase tunability of parametric system-bath interactions.

Further improvements of the proposed scheme using simple design variations, such as using parametric qubit-qubit drives instead of direct drives (Supplementary Note 3) and a moderate increase in resonator linewidth possibly coupled with the addition of a Purcell filter^41^, can lead to 8–10% higher fidelity with current hardware. Since most of the state preparation error is due to the residual ground state population, a straightforward improvement in fidelity is achievable by using the center resonator to herald based on the state parity^6,15^. While the present work relies on time-independent drive parameters only, a combination of approach presented here with time-dependent control explored previously in the context of approximate stabilization schemes^40,42^ can be an attractive avenue to do a fully optimized protocol that mitigates both intrinsic and (dephasing-induced) extrinsic errors. We expect that suppression of intrinsic error, as demonstrated here, can ease the implementation, characterization, and optimization of stabilization protocols: such considerations will become increasingly important when considering stabilization of larger and more complex states. The design principles underlying this work thus provide a novel addition to the parametric toolbox for quantum control in systems with strong light-matter interactions and can be readily extended for stabilization of multi-partite entangled states in large quantum networks.

## Methods

### Simulation approach

We simulated our scheme using the following Lindblad master equation:

$$\dot{\rho } =	 -i[{H}_{I},\rho ]+\kappa {{{{{{{\mathcal{D}}}}}}}}[a]\rho \\ 	+\mathop{\sum}\limits_{k\in \{l,r\}}\left({\gamma }_{k}^{01}{{{{{{{\mathcal{D}}}}}}}}[{\left|0\right\rangle }_{k}\left\langle 1\right|]+{\gamma }_{k}^{12}{{{{{{{\mathcal{D}}}}}}}}[{\left|1\right\rangle }_{k}\left\langle 2\right|]\right.\\ \,	+\left.2{\gamma }_{k}^{11}{{{{{{{\mathcal{D}}}}}}}}[{\left|1\right\rangle }_{k}\left\langle 1\right|]+2{\gamma }_{k}^{22}{{{{{{{\mathcal{D}}}}}}}}[{\left|2\right\rangle }_{k}\left\langle 2\right|]\right)\,\rho ,$$

where ${{{{{{{\mathcal{D}}}}}}}}[o]\rho =o\rho {o}^{{{{\dagger}}} }-\frac{1}{2}\{{o}^{{{{\dagger}}} }o,\rho \}$ and *H*_*I*_ denotes the interaction Hamiltonian in Eq. (1). In order to simulate pump amplitude-dependent shifts, we also include Hamiltonian terms of the form ${\sum }_{k = l,r}{\delta }_{k}^{01}{\left|1\right\rangle }_{k}\left\langle 1\right|$ and *δ*_*a*_*a*^†^*a* that describe qubit and resonator detunings respectively. The measured relaxation rates are ${\gamma }_{l,r}^{01}$ and ${\gamma }_{l,r}^{12}$. The pure dephasing rates for $\left|0\right\rangle +\left|1\right\rangle$ and $\left|0\right\rangle +\left|2\right\rangle$ are ${\gamma }_{l,r}^{11}$ and ${\gamma }_{l,r}^{22}$ respectively. We have assumed that relaxation is a sequential process 2 → 1 → 0 and cross-dephasing terms can be ignored^43^. The latter approximation is justified since dephasing in our device is primarily due to thermal photons in the resonator. Moreover, we neglect the 0–2 decay process as it is a forbidden transition as per selection rules of the transmon. Detailed list of experimental parameters used for performing master equation simulations is included in Supplementary Table 1. For performing simulations, the absolute and relative tolerances of the 12th-order Adams-Moulton solver in QuTiP^44^ are each set to 10^−12^. Further, we truncate the Hilbert space corresponding to a maximum photon number *n* = 6 in the resonator, beyond which we do not observe any appreciable change in the simulated Liouvillian gap with the number of levels.

### Semi-classical estimate of steady-state error vs. convergence time

As our protocol has no intrinsic error process, the steady state stabilization error is set primarily by competition between the stabilization process pumping population into the target Bell state $\left|T\right\rangle$ and local decoherence leading to decay from the target state. In the absence of decoherence, the system would relax into the target state exponentially at a rate *τ*^−1^, so $\dot{\varepsilon }(t) \sim -{\tau }^{-1}\varepsilon (t)$. If instead the stabilization mechanism were turned off and we consider only the effect of decoherence, at short times the decay out of $\left|T\right\rangle$ is exponential with a rate $\gamma \approx {T}_{B}^{-1}$ leading to $\dot{F}(t)=-\dot{\varepsilon }(t) \sim -{T}_{B}^{-1}(1-\varepsilon (t))$. Taking both these processes together, we estimate the steady state error *ε*_*∞*_ by solving $\dot{\varepsilon }(t)=0$, yielding *ε*_*∞*_ ≈ *τ*/*T*_*B*_, when in the dissipation engineering regime where *τ* ≪ *T*_*B*_. As shown in Supplementary Fig. 11, this estimate accurately predicts the simulated steady state error of the protocol in the absence of detuning- or asymmetry-driven coherent error processes.

### Comparison to other stabilization schemes

The estimated ${T}_{2}^{* }=100$ μs in Table 1 for the trapped ions stabilization scheme (ref. ^17^) is based on assuming magnetic field fluctuation amplitude of 0.1 μT and magnetic field sensitivity of 17.6 kHz/μT for hyperfine states $\left|1,1\right\rangle ,\left|2,2\right\rangle$ in ^9^*Be*^+^. This estimate is also consistent with other values of ${T}_{2}^{* }=80$ μs reported in ref. ^45^ for a different pair of levels, $\left|1,-1\right\rangle ,\left|2,-2\right\rangle$, with slope 21 kHz/μT. Note that ref. ^17^ also implemented a step-wise version of their stabilization protocol which further reduces the impact of the qubit dephasing rate, allowing them to achieve 89% fidelity in 30 steps of 220 μs each. This stepped implementation is less directly comparable to the other CW schemes. Also ref. ^15^ presented an additional result using post-selection, allowing them to slightly increase the measured fidelity of their CW protocol to 77%.

## Supplementary information

Supplementary Information

## Data Availability

The data that support the findings of this study are available from the corresponding author upon reasonable request.
