# Supplementary material for: Trade off-free entanglement stabilization in a superconducting qutrit-qubit system
Source: Nat Commun. 2022 Jul 9;13:3994. doi: 10.1038/s41467-022-31638-0 (PMC9271051; doi:10.1038/s41467-022-31638-0)
Supplement: Supplementary file 1 — Supplementary Information [file 41467_2022_31638_MOESM1_ESM.pdf]

# Supplementary Information

# Trade off-Free Entanglement Stabilization in a Superconducting Qutrit-Qubit System

T. Brown,[1,2] E. Doucet,[1] D. Ristè,[2] G. Ribeill,[2] K. Cicak,[3]
J. Aumentado,[3] R. Simmonds,[3] L. Govia,[2] A. Kamal,[1] and L. Ranzani*[2]

[1]*Department of Physics and Applied Physics, University of Massachusetts, Lowell, MA 01854, USA*
[2]*Quantum Engineering and Computing, Raytheon BBN Technologies, Cambridge, MA 02138, USA*
[3]*National Institute of Standards and Technology, 325 Broadway, Boulder, CO 80305, USA*
(Dated: June 20, 2022)

## SUPPLEMENTARY NOTE 1: CIRCUIT HAMILTONIAN

The circuit is described by the following Hamiltonian,

$$H = \omega_a a^\dagger a + \sum_{k\in\{l,r\}} \left(\omega_k b_k^\dagger b_k + \frac{\alpha_k}{2} b_k^\dagger b_k (b_k^\dagger b_k + 1)\right) \\ + \sum_{k\in\{l,r\}} g_k(\Phi(t))(b_k + b_k^\dagger)(a + a^\dagger) + \sum_{k\in\{l,r\}} \Omega_k(t)(b_k + b_k^\dagger), \tag{1}$$

where $\omega_k^{n,n+1} = \omega_k + (n+1)\alpha_k$ define the transition frequencies of the transmon $k \in \{l, r\}$, and $\omega_a$ denotes the center frequency of the resonator. $\Omega_k(t)$ is the amplitude of the direct drives on the transmons. The SQUID mediates a tunable coupling strength, $g_k(\Phi) = L_{sq}(\Phi)\sqrt{\omega_k\omega_a}/(2\sqrt{L_k L_a})$, between each transmon and the resonator, where $L_{sq}$ denotes the flux-tunable coupler inductance and $L_k$ is the inductance of the respective transmon [1, 2]. We assume that $\Phi(t)$ and $\Omega_k(t)$ are periodic functions of time, so that:

$$g_k(t) = \sum_{k\in\{l,r\}} \left(g_k^{01}\cos(\omega_{pk}^{01}t + \phi_k^{01}) + \frac{g_l^{12}}{\sqrt{2}}\cos(\omega_{pl}^{12}t + \phi_l^{12})\right) \tag{2}$$

$$\Omega_k(t) = \sum_{k\in\{l,r\}} \Omega_k^{01}\cos(\omega_{dk}^{01}t + \theta_k). \tag{3}$$

We can then eliminate the time dependence in the Hamiltonian by moving to a rotating frame w.r.t. the drives. We write $b_k = \sum_j \sqrt{j+1}|j\rangle_k\langle j+1|$ and perform a unitary transformation described by

$$U(t) = \exp\left[-i\left(\Delta_a a^\dagger a + \Delta_r^{(1)}|1\rangle_r\langle 1| + \Delta_l^{(1)}|1\rangle_l\langle 1| + \Delta_l^{(2)}|2\rangle_l\langle 2|\right)t\right] \tag{4}$$

to move into a rotating frame with respect to the free Hamiltonian [first line in Supplementary Eq. (1)]. Keeping only the co-rotating terms for the choice of parametric drive frequencies $\omega_{pk}^{01} = \Delta_a - \Delta_k^{(1)}$, $\omega_{pl}^{12} = \Delta_a - \Delta_l^{(2)}$ and Rabi drive frequencies $\omega_{dk}^{01} = \Delta_k^{(1)}$, we obtain,

$$H' = U^\dagger H U - i\hbar U^\dagger \frac{dU}{dt} \\ = \delta_a a^\dagger a + \sum_{k\in\{l,r\}} \delta_k^{01}|1\rangle_k\langle 1| + \delta_l^{12}|2\rangle_l\langle 2| + H_\mathrm{I}, \tag{5}$$

with

$$H_\mathrm{I} = a^\dagger\left(\frac{g_l^{12}}{2}e^{i\phi_l^{12}}|1\rangle_l\langle 2| + \sum_{k\in\{l,r\}}\frac{g_k^{01}}{2}e^{i\phi_k^{01}}|0\rangle_k\langle 1|\right) + \sum_{k\in\{l,r\}}\frac{\Omega_k^{01}}{2}e^{i\theta_k}|0\rangle_k\langle 1| + h.c., \tag{6}$$

Here $\delta_a = \Delta_a - \omega_a$ is the resonator detuning, $\delta_k^{01} = \Delta_k^{(1)} - \omega_k^{01}$ denote the detunings from 0-1 transitions frequencies, and $\delta_l^{12} = \Delta_l^{(2)} - \omega_l^{12}$ is the detuning from 1-2 transition of the qutrit.

In arriving at this Hamiltonian, we chose to consider only the low three levels of the left transmon and the low two levels of the right transmon, giving us an asymmetric qutrit-qubit system. As discussed in the main text and expanded

upon in the next section, this is the *minimal* extension to the system Hilbert space which allows exact stabilization of a Bell state. We could instead have chosen to truncate both transmons to the low three levels and work with a qutrit-qutrit system instead. Our scheme generalizes to this case with the addition of another red-sideband drive on either the 1-2 and 0-2 transitions of the right transmon to ensure any population in the second excited state decays quickly into the qubit manifold.

## SUPPLEMENTARY NOTE 2: MINIMALITY OF QUTRIT-QUBIT SCHEME

In this section we motivate why our scheme is minimal to achieve trade off-free stabilization of a two-qubit Bell state. In dissipative stabilization, a quantum system is coupled to its environment so that the desired target state is a stationary state of the dynamics, forming a so-called dark state. The conditions [3] for the target state $|\psi\rangle$ to be a dark state are: (i) $|\psi\rangle$ is an eigenstate of the system Hamiltonian, and (ii) $|\psi\rangle$ is not a left eigenstate of the jump operator, i.e.

$$L|\psi\rangle = 0 \quad \text{and} \quad \langle\psi|L \neq 0 \tag{7}$$

The first condition in (7) prevents decay *out of* the target state, while the second condition states that the target state is *not* a dark state of the dual (time-reversed) system, *i.e.* there is a decay channel *into* the target state. The latter is necessary to ensure that the desired state can be reached irrespective of the initial conditions of the system. Restricting to only quasi-local jump operators that are realizable with red- and blue-sideband couplings, the most general form of $\hat{L}$ is,

$$L = \left(c_1^- \sigma_1 + c_2^- \sigma_2 + c_1^+ \sigma_1^\dagger + c_2^+ \sigma_2^\dagger\right), \tag{8}$$

where $c_j^\pm$ are arbitrary coefficients set by the amplitudes and phases of the sideband drives. Satisfying the constraints in Supplementary Eq. (7) for $|\psi\rangle = |S\rangle = (1/\sqrt{2})(|01\rangle - |10\rangle)$ requires that the sideband drive parameters be chosen such that $c_1^\pm = c_2^\pm$. Unfortunately, this immediately implies that the second constraint in Supplementary Eq. (7) is impossible to satisfy due to resultant symmetry of the evolution operators as explained in the main text.

Typical Bell state stabilization protocols employing linear dissipation circumvent this issue by 'dressing' the target state. If the target state is not chosen to be the exact singlet state but $(|S\rangle + \delta|\xi\rangle)/\sqrt{1+\delta^2}$ instead, where $|\xi\rangle$ is orthogonal to $|S\rangle$ (e.g. $|gg\rangle$ [4]), then it is possible to find a set of $c_j^\pm$ for which both constraints in Supplementary Eq. (7) can be satisfied simultaneously. Note that $\delta$ needs to be small, for the singlet fraction in the ideal steady-state to be high,

$$F_{ss}(\delta) = |\langle S|\psi\rangle|^2 = \frac{1}{1+\delta^2} \sim 1 - \delta^2. \tag{9}$$

However, in the limit of $\delta \to 0$ we find that the stabilization rate $\tau^{-1} \to 0$ also. To see this, note that by Fermi's golden rule, the decay rate into $|\psi\rangle$ from $|\phi_j\rangle$ is proportional to the magnitude squared of the matrix element $\langle\psi|L|\phi_j\rangle$. Since $L^\dagger|S\rangle = 0$, this must be proportional to $\delta$,

$$\langle\psi|L|\phi_j\rangle = \frac{\delta}{\sqrt{1+\delta^2}}\langle\xi|L|\phi_j\rangle \sim \delta\langle\xi|L|\phi_j\rangle. \tag{10}$$

These two results taken together force a trade off to be made when operating a protocol based on this approach; thus achieving high fidelity requires that $\delta$ be as small as possible, but this necessarily causes the stabilization rate to drop. In particular the product between error $1 - F_{ss}$ and time $\tau$ is approximately constant.

In practice, the impact of this trade off depends on the parameter regime the protocol is operated in. When stabilizing a Bell state with such a scheme, we can identify two conceptually distinct sources of error in the late time limit. To this end, we should expect the late-time error to scale as

$$\begin{aligned}
\varepsilon(t \to \infty) &= 1 - \mathrm{Tr}\left[\rho(t \to \infty)|S\rangle\langle S|\right] \\
&= 1 - \langle S|\big((1-\varepsilon_\gamma)|\psi\rangle\langle\psi| + \varepsilon_\gamma \rho^\perp\big)|S\rangle \\
&\approx 1 - |\langle S|\psi\rangle|^2 (1-\varepsilon_\gamma) \\
&\equiv 1 - (1-\varepsilon_\delta)(1-\varepsilon_\gamma) = \varepsilon_\delta + \varepsilon_\gamma - \varepsilon_\delta\varepsilon_\gamma,
\end{aligned} \tag{11}$$

From Supplementary Eq. (11), it is clear that the two sources of error limiting the terminal fidelity are:
(i) "extrinsic error", $\varepsilon_\gamma$, due to various decoherence processes that affect the qubits preventing unit fidelity stabilization of the state $|\psi\rangle$,
(ii) "intrinsic error", $\varepsilon_\delta$, due to the target state $|\psi\rangle$ itself not being an exact Bell state.

The error $\varepsilon_\gamma = \gamma/\Gamma$ can be approximated as a ratio of net decay out of the target state due to decoherence ($\gamma$) and the net rate of condensation into the target state due to stabilization ($\Gamma$); this follows from the construction used in the main text to find the theoretical limit of error-time scaling using a simple rate equation model. The existence of a trade off ensures that both $\varepsilon_\delta$ and $\Gamma$ vary with $\delta$, and that for any given implementation with fixed decay rates and fixed maximum drive strengths, there exists some $\delta$ which optimizes the overall fidelity. Typically, we expect that the parameters which minimize Supplementary Eq. (11) should lead to an approximate balance between the different sources of error, $\varepsilon_\delta \approx \varepsilon_\gamma$. This is in line with the results obtained when optimizing detunings for dephasing mitigation in [5].

On the other hand, the protocol demonstrated in this work realizes a reservoir engineering scheme which has, in principle, no intrinsic error, i.e. $\varepsilon_\delta = 0$. This alleviates the trade off imposed by the interplay of $\varepsilon_\delta$ and $\Gamma$, leading to a low total error (now entirely limited by $\varepsilon_\gamma$) *and* a large stabilization rate $\Gamma$. In a recent work [6], we theoretically showed that this is possible by employing a strong dispersive coupling between the qubits and the auxiliary cavity to lift the energy degeneracy between the zero and one photon manifold of the qubit-cavity system, thus expanding the set of accessible jump operators.

The scheme presented in the main text uses a different approach with unconditional interactions and linear dissipation, expanding the Hilbert space to include the second excited state of one of the transmons. This is the smallest extension of the two-qubit Hilbert space possible, but it is enough such that quasi-local dissipation in this larger Hilbert allows the use of unconditional CW interactions to achieve Bell state stabilization without any trade off between fidelity and speed. This is illustrated in Fig. (2b) of the main text, which shows how the stabilization mechanism of the scheme works by using the extra qutrit levels to allow an indirect path from the triplet manifold into the singlet manifold. The effective Hamiltonian $H$ and jump operator $L$ after adiabatic elimination of the resonator are illustrated schematically in Supplementary Fig. 1. Note the off-diagonal elements of $L^\dagger L$, which indicate that the transmon-resonator coupling induces coherent interactions between the two transmons, in addition to implementing dissipation on different two-qubit states.

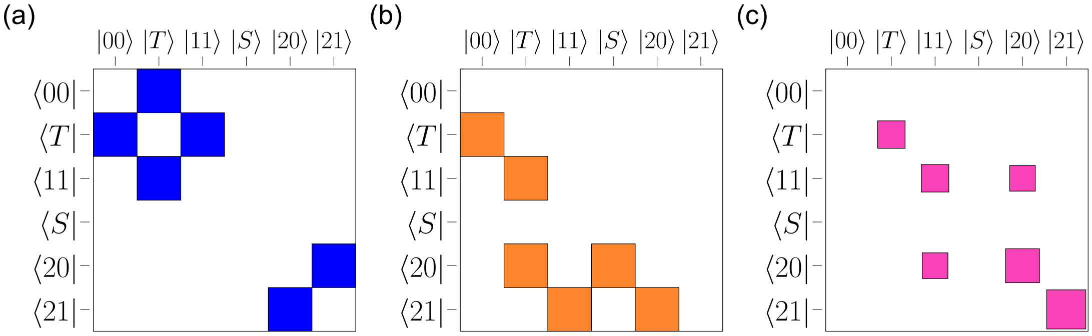


FIG. 1. Hinton diagrams of the effective (a) $H$, (b) $L$, and (c) $L^\dagger L$ in the qutrit-qubit space after adiabatic elimination of the resonator.

A salient advantage of using higher levels instead of dispersive shifts to implement state-selective driving is the diminished effect of counter-rotating terms that become relevant at strong drive amplitudes. These terms beyond the rotating-wave approximation have a negative impact on the performance of the stabilization scheme by introducing coherent leakage out of the target state. The dominant counter-rotating contributions in our stabilization scheme are described by adding the following terms to the interaction Hamiltonian,

$$H_{\mathrm{CR}} = a^\dagger \left( \frac{g_l^{12}}{2\sqrt{2}} e^{i\phi_l^{12}} e^{-i\alpha_l t} |0\rangle_l \langle 1| + \sum_{k=l,r} \frac{g_k^{01}}{\sqrt{2}} e^{i\phi_k^{01}} e^{+i\alpha_k t} |1\rangle_k \langle 2| \right) + \sum_{k\in l,r} \frac{\Omega_k^{01}}{\sqrt{2}} e^{i\theta_k} e^{+i\alpha_k t} |1\rangle_k \langle 2| + h.c. \tag{12}$$

As it is evident in the above formula, the induced leakage is off-resonant by the anharmonicities $\alpha_{l,r}$ of the transmons, which is at least an order of magnitude larger than typical dispersive shift-induced leakage in protocols using number-selective [6] or state-selective driving [7]. Accordingly, as shown in Supplementary Fig. 2, these terms are negligible even for parametric drive amplitudes in excess of 10 MHz.

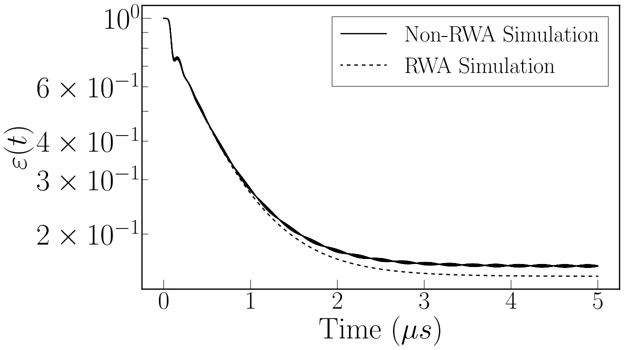


FIG. 2. Comparison of stabilization performance using RWA Hamiltonian (Eq. (1) in the main text) and simulations including Supplementary Eq. (12). The average steady-state fidelity changes from 85.2% to 84.0% upon including counter-rotating terms, a small change which we expect to be within the margin of measurement and reconstruction error.

## SUPPLEMENTARY NOTE 3: EXTENSIONS TO THE STABILIZATION SCHEME

While not used in the present work, our device design allows parametric transmon-transmon interactions in addition to the parametric transmon-resonator interactions employed for dissipation engineering. In fact it is in principle possible to design a purely parametric stabilization protocol according to the following Hamiltonian:

$$H = \omega_a a^\dagger a + \sum_{k\in\{l,r\}} \left(\omega_k b_k^\dagger b_k + \frac{\alpha_k}{2} b_k^\dagger b_k (b_k^\dagger b_k + 1)\right) \tag{13}$$

$$+ \sum_{k\in\{l,r\}} g_k(\Phi(t))(b_k + b_k^\dagger)(a + a^\dagger) + g_{qq}(\Phi(t))(b_l + b_l^\dagger)(b_r + b_r^\dagger), \tag{14}$$

where additional flux drives are included such that $g_{qq}(t)$ can drive transmon-transmon interactions on resonance. In particular our scheme can be made purely parametric by use of a transmon-transmon blue-sideband drive at $\omega_{lr}^+ = \omega_l^{01} + \omega_r^{01}$ in lieu of the direct transmon drives in the present scheme, yielding a purely parametric stabilization scheme with several advantages over the version built around direct driving. The effective interaction Hamiltonian for such a scheme is,

$$H_\mathrm{I} = a^\dagger \left( \frac{g_l^{12}}{2} e^{i\phi_l^{12}} |1\rangle_l\langle 2| + \sum_{k\in\{l,r\}} \frac{g_k^{01}}{2} e^{i\phi_k^{01}} |0\rangle_k\langle 1| \right) + \frac{g_{qq}}{2} |00\rangle\langle 11| + h.c. \tag{15}$$

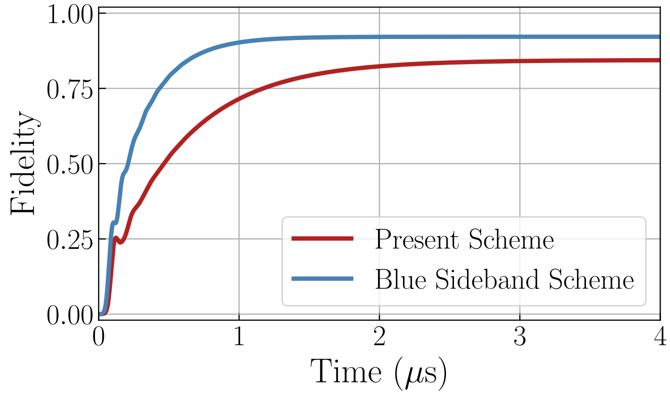


FIG. 3. Comparison of stabilization trajectories for current direct drive-based stabilization scheme (red) and a scheme utilizing a two-photon parametric drive on qubits (blue). In both cases, the transmon resonator couplings were set to $g_{l,r}^{01} = 2\pi \times 7.5\,\mathrm{MHz}$ and $g_l^{12} = 2\pi \times 13.1\,\mathrm{MHz}$. For the present scheme, the optimal direct drive strength used is $\Omega_{l,r} = 2\pi \times 7.2\,\mathrm{MHz}$. For the two-photon driving scheme, the optimal coupling strength used is $g_{qq} = 2\pi \times 15.2\,\mathrm{MHz}$.

Using exactly the same sideband coupling strengths and an optimized transmon-transmon coupling, we would expect to achieve a fidelity of 92%, as compared to 84% achieved using direct drives, on the same device as illustrated by the simulations presented in Supplementary Fig. 3. Such two-photon interaction was not available in our device, because stray capacitive coupling between the flux line and the SQUID limited the available bandwidth, but it should be accessible with relatively straightforward design modifications. Further optimization of transmon-resonator coupling strengths over a wide range of parameters (including the case when the transmon-transmon drive is comparable in strength to the direct drive strengths currently used), we expect a 2×-3× improvement in both stabilization time and error from the inclusion of parametric qubit-qubit interactions.

## SUPPLEMENTARY NOTE 4: EXPERIMENTAL SETUP AND CHARACTERIZATION

The experimental setup is shown in Supplementary Fig. 4. The sample is enclosed in an Aluminum box, wirebonded to coaxial connectors and mounted at the 20mK stage of a dilution refrigerator. Qubit readout and control signals are injected via a single heavily attenuated microwave line and the reflected qubit readout signals are amplified, demodulated and integrated with a matched filter [8]. The parametric drives are combined, attenuated by 42dB and lowpass filtered before reaching the SQUID flux port. The 4 GHz lowpass filter at base temperature protects the qubits from noise and decay through the pump line. We further minimized the length of coaxial cables between the device and filter stages to avoid standing wave resonances near the qubit frequencies. The qubit device is further enclosed into a $\mu$-metal shield to protect it from stray magnetic fields.

The SQUID flux bias signal is lowpass filtered via a 3-stage RC network at 4 K and an Ecosorb filter at base temperature. We used a multi-channel signal generator for the control and parametric drives to guarantee relative phase stability. The microwave tones are then sideband modulated by fast arbitrary waveform generators [8] to obtain the desired signal frequencies.

### Device Characterization

We characterized the 0-1 and 1-2 transition frequencies for transmons using pulsed microwave spectroscopy [Fig. 1 of the main text], and extracted Josephson energy $E_J$ and charging energy $E_c$ [9] of the junctions using standard formulae. Furthermore, we measured the relaxation and dephasing rates of the three lowest transmon levels by performing inversion recovery and Ramsey experiments [10]. For the $1-2$ transition we verified that the $\gamma^{12} \approx 2\gamma_{01}$ in accordance with the dipole matrix element for a transmon-type oscillator, by fitting the measured decay curves for the three levels to a sequential decay process $2 \to 1 \to 0$. To extract the dephasing time for $0-2$ and $1-2$ transitions, we first subtracted exponential background decay to level $|1\rangle_k$ and then fitted the measured oscillations to a decaying sinusoid. The device parameters are summarized in Table I.

| Parameter | **Measured Value** |
|---|---|
| Josephson Energy ($E_J/2\pi$) | $l = 24.4$ GHz, $r = 20.9$ GHz, SQUID $\approx 340$ GHz |
| Anharmonicity ($\alpha_{l,r}/2\pi$) | $l = -198$ MHz, $r = -164$ MHz |
| Rabi drive amplitude ($\Omega^{01}_{l,r}/2\pi$) | 7.2 MHz |
| 0-1 sideband amplitude ($g^{01}_{l,r}/2\pi$) | 7.5 MHz |
| 1-2 sideband amplitude ($g^{12}_{l}/2\pi$) | 13.1 MHz |
| Resonator decay rate ($\kappa/2\pi$) | 4.73 MHz |
| 0-1 relaxation rate ($\gamma^{01}/2\pi$) | $l = 33.9$ kHz, $r = 27.5$ kHz |
| 1-2 relaxation rate ($\gamma^{12}/2\pi$) | $l = 69.2$ kHz $r = 56.9$ kHz |
| 0-1 dephasing rate ($\gamma^{11}/2\pi$) | $l = 18.8$ kHz, $r = 13.9$ kHz |
| 0-2 dephasing rate ($\gamma^{22}/2\pi$) | $l = 43$ kHz $r = 32.8$ kHz |

TABLE I. Summary of measured device parameters.

The envelope of the Ramsey oscillations is exponential indicating that dephasing is dominated by white noise. Under the assumption that this white noise component was due entirely to photon shot noise in the center resonator, we can infer the (residual) photon population $\langle n \rangle$ in the resonator in the weak dispersive regime (since $\chi/\kappa < 1$); using $\gamma^{11} = 8\langle n \rangle \chi^2/(\kappa + 4\chi^2/\kappa)$ [11], we obtain an average photon number $\langle n \rangle \approx 0.02$ consistent with thermal populations

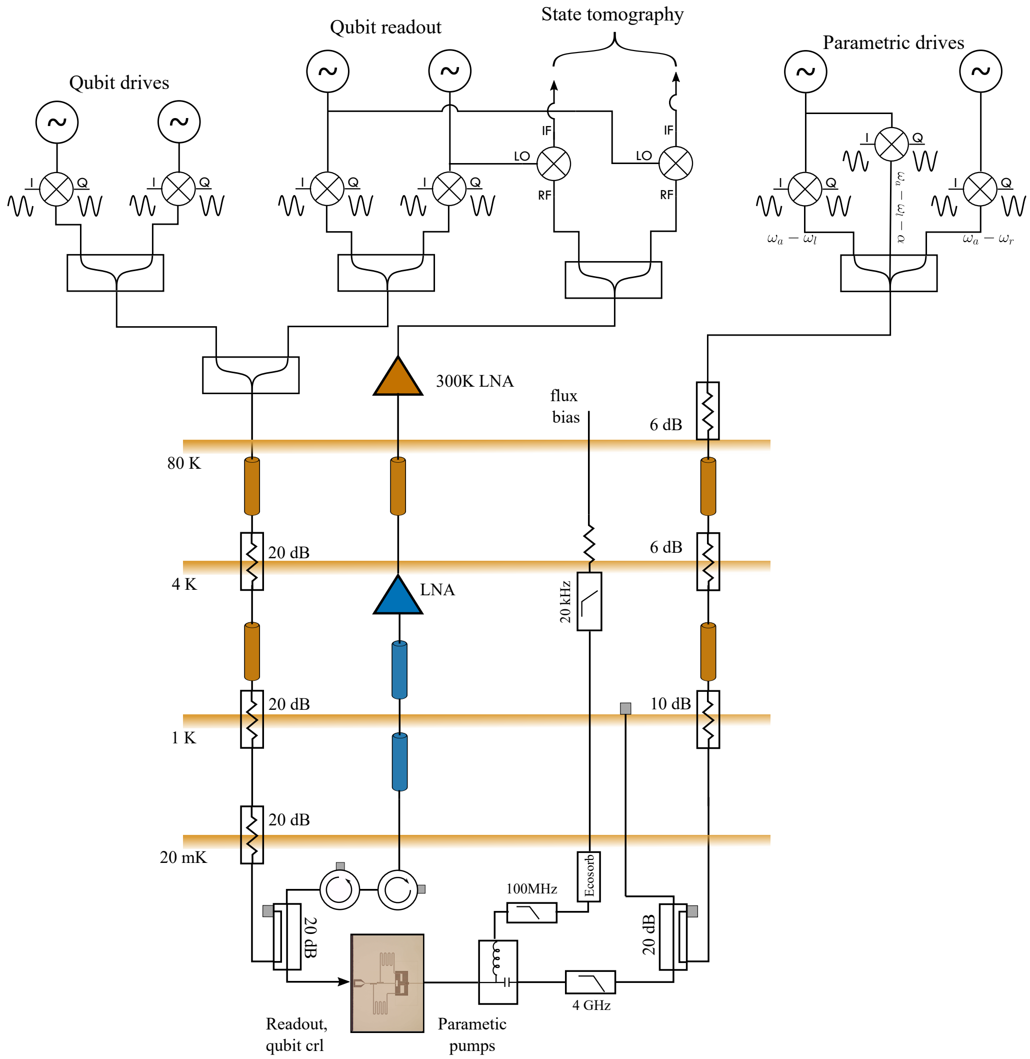


FIG. 4. Experimental Setup

reported previously in other cQED setups. In the absence of correlated noise (for instance, $1/f$-type), the dephasing rate of the 1-2 transition can be approximated as $\gamma_\phi^{12} \approx \gamma^{22} + \gamma^{11}$ [12]; this describes the dephasing of 1-2 Ramsey oscillations shown in Supplementary Fig. 5.

**Stabilization Drives Tuning Procedure**

Here we describe our procedure to tune the stabilization drives together to compensate for pump-induced Stark shifts of the qubit and resonator frequencies due to the SQUID nonlinear inductance [13]. Most tuning steps require only knowledge of the diagonal part of the density matrix, which we extracted directly from expectation values of $\langle \sigma_{zl,r} \rangle$ and the correlator $\langle \sigma_{zl}\sigma_{zr} \rangle$ (see section on Quantum State Tomography later).

We initially calibrate each drive independently as explained in the main text and use the resulting frequencies and amplitudes as our starting point. We then initialize the system in either $|01\rangle$ or $|10\rangle$ and apply the parametric drives for a time $t \gg 1/\kappa$, but much lower than the qubit decoherence times, see Supplementary Fig. 6(a). We verified from

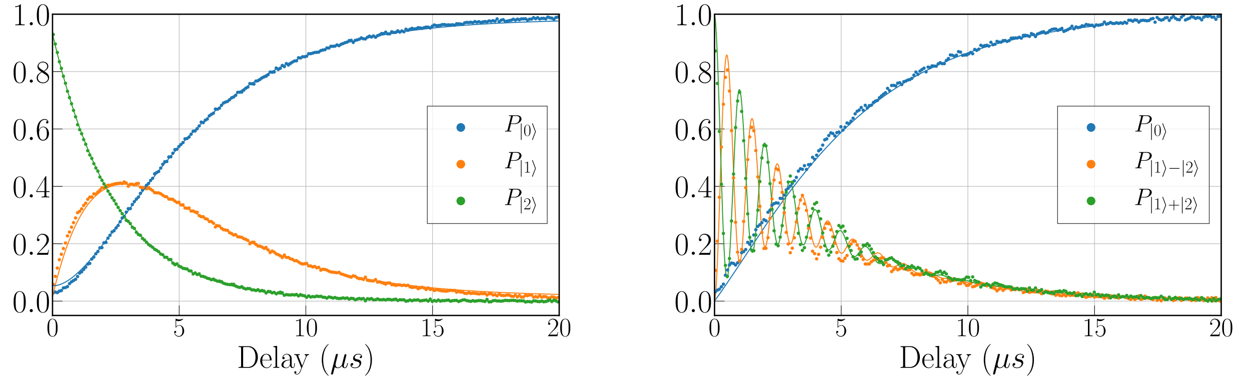


FIG. 5. Left: Populations in $|0\rangle$, $|1\rangle$ and $|2\rangle$ in a transmon qutrit prepared in $|2\rangle$ and allowed to relax. Population in $|0\rangle$ deviates from the data due to measurement errors leading to a slightly reduced trace. Right: $|0\rangle$, $(|1\rangle - |2\rangle)/\sqrt{2}$ and $(|1\rangle + |2\rangle)/\sqrt{2}$ populations tracked over time in a Ramsey experiment with the transmon qutrit initialized in $(|1\rangle + |2\rangle)/\sqrt{2}$. In both plots, experimental data is shown as dots and master equation simulations are shown as lines.

our simulations that at such time the population in the even parity manifold reaches a minimum when the drives are on resonance, allowing us to determine each drive frequency Supplementary Fig. 6(c)]. Moreover, the ratio $P_{10}/P_{01}$ between the qubit state populations quickly converges toward the ratio $(g_r^{01}/g_l^{01})^2$ consistent with the steady state in the presence of asymmetric 0-1 sideband amplitudes,

$$\rho_{ss} = \alpha|00\rangle\langle 00| + \beta|\psi_{\text{odd}}\rangle\langle\psi_{\text{odd}}|, \tag{16}$$

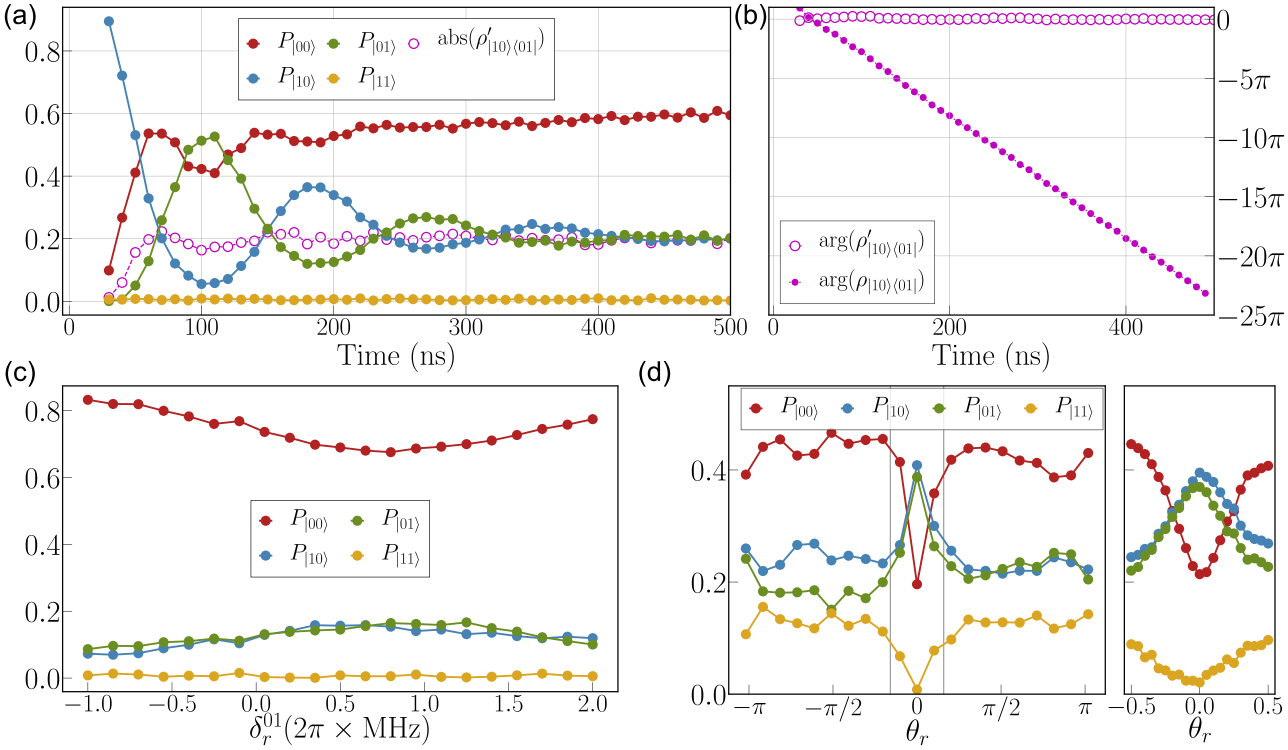


FIG. 6. (a) Evolution of the state populations when initializing in $|10\rangle$ under just parametric drives. $P_{|00\rangle}$ is minimized when the drives are on resonance. (b) AC Stark shift of the qubits introduces a Z-rotation of the state $\rho$. Such rotation is compensated by adjusting the drive phases, resulting in the state $\rho'$. (c) Effect of parametric drive detuning on $P_{|00\rangle}$. (d) When the Rabi drives are introduced the Rabi and parametric drive phases need to be aligned to stabilize the Bell state. When the phases are aligned the qubit population in the even-parity manifold is minimized.

where $|\psi_{\rm odd}\rangle = (g_r^{01}|10\rangle - g_l^{01}|01\rangle)/\sqrt{(g_l^{01})^2 + (g_r^{01})^2}$. This allows fine tuning of 0-1 sideband amplitudes by minimizing the difference between $P_{10}, P_{01}$ in the steady state. Note that while the steady state of the system in the presence of all stabilization drives is independent of the initial state of the qubits, this is not the case in the absence of Rabi drives.

At this point of the tuning process the third parametric drive at $\omega_a - \omega_l^{12}$ affects the qubit and resonator frequencies but does not produce any transition, because of photon number conservation. In order to tune the third parametric drive, we initialize the qubits in $|20\rangle$, so that the $|20\rangle|0\rangle_a$ to $|10\rangle|1\rangle_a$ swap process can be observed. We subsequently minimize the expectation value of $\langle\sigma_{zr}\rangle$ as a function of the drive frequency at $t \approx 150\,\text{ns}$, which corresponds to a population transfer between the transmons with maximum efficiency. Once all parametric drives are tuned on resonance, we perform full state tomography of the qubits over time and measure the state $Z$-rotation caused by the pump-induced AC Stark shift, see Supplementary Fig. 6(b). Such $Z$-rotation is computed accurately from the time evolution of the phase of the $\langle 10|\rho(t)|01\rangle$ element of the density matrix and it is equal to $\delta_s t$, where $\delta_s = \delta_l^{01} + \delta_r^{01}$ is the sum of the qubit frequency shifts.

We finally proceed to tune the Rabi drives. From the previous steps we know the Rabi drive common-mode frequency offset $\delta_s$ as well as their differential offset, since the latter has to match the relative frequency offset of the $g_{l,r}^{01}$ parametric drives. We then proceed to sweep the Rabi drives relative phase and amplitudes until the population of the desired Bell state $|T\rangle$ reaches a maximum, see Supplementary Fig. 6(d). Finally, we further compensate the state $Z-$rotation by adjusting the phase of the parametric pumps and Rabi drives as a function of the pulse width. For long stabilization times we observe a small residual detuning of the order of 50 kHz, which we correct in post-processing [Supplementary Fig. 6(b)].

## Quantum State Tomography

We used Quantum State Tomography (QST) to characterize the stabilized state [14]. We repeatedly prepared the stabilized state by turning on the stabilization drives for a fixed amount of time and then performed a set of single qubit rotations followed by simultaneous readout of both qubits with separate cavities, with readout fidelity of 86%. The tomographic gate set consisted of idle, $X_\pi$, $X_{\pm\pi/2}$ and $Y_{\pm\pi/2}$ gates on each qubit, for a total of 36 gate combinations. While the minimal gate set for two-qubit QST consists of 16 gate combinations [15, 16], 36 gate combinations provide an overdetermined set of equations that reduce reconstruction errors. We then measured the expectation values of single-qubit polarization $\langle Z_k\rangle = |0\rangle_k\langle 0| - |1\rangle_k\langle 1|$, as well as the product $\langle Z_l Z_r\rangle$ by correlating the single measurement shots from each qubit and averaging. For each tomographic measurement we averaged over $8\times 10^4$ records. We used a least squares optimizer, weighed by the variance of the observations, to reconstruct the density matrix. The optimizer minimizes the quantity [14]

$$E(\rho) = \sum (M_j - P_j(\rho))^2/var(M_j), \tag{17}$$

where $M_j$ are the measured single and two-body observables and $P_j$ are the predicted values for a two-qubit state defined by he density matrix $\rho$. Weighing by the variance of the data reduces bias in the reconstructed matrix elements by taking into account that the errors in the measured single- and two-body terms have different standard deviation.

*Approximate calculation of qubit populations* - To tune up the drives we compute only the diagonal elements of the density matrix, without performing a full tomographic reconstruction. The diagonal elements are in fact directly affected by both amplitude imbalance or frequency detuning of the drives, as detailed in the previous section on tuning. We can compute the diagonal elements of $\rho$ directly by measuring $\langle Z_l\rangle$, $\langle Z_r\rangle$ and $\langle Z_l Z_r\rangle$ and directly computed the population of the four basis states $|00\rangle$, $|01\rangle$, $|10\rangle$, $|11\rangle$ as,

$$P_{00} = \frac{I + \langle Z_l\rangle + \langle Z_r\rangle + \langle Z_l Z_r\rangle}{4},$$
$$P_{01} = \frac{I + \langle Z_l\rangle - \langle Z_r\rangle - \langle Z_l Z_r\rangle}{4},$$
$$P_{10} = \frac{I - \langle Z_l\rangle + \langle Z_r\rangle - \langle Z_l Z_r\rangle}{4},$$
$$P_{11} = \frac{I - \langle Z_l\rangle - \langle Z_r\rangle + \langle Z_l Z_r\rangle}{4}.$$

Note that because of the crosstalk between the two measurement channels, the measurement records are in general a linear combination of $Z$-measurement operators. We calibrated and removed cross-talk between the readout signals by measuring the readout levels for all four basis states and performing a linear inversion.

### Stabilization from Different Initial States

One of the features of our dissipative stabilization protocol is that it is initial state-independent by construction. Any initial state will relax to the engineered steady state of the resulting Liouvillian, allowing for universal stabilization. This is possible because every state in the two-qubit Hilbert space is coupled to the target state via a combination of fast coherent and dissipative channels. This is demonstrated for the stabilization scheme implemented in the main text in Supplementary Fig. 7. Note that when the qubits are initialzied in any of the four basis states, the system converges to the same target state with similar fidelity. Moreover an important feature of our protocol is that the convergence time is in fact independent from the initial state.

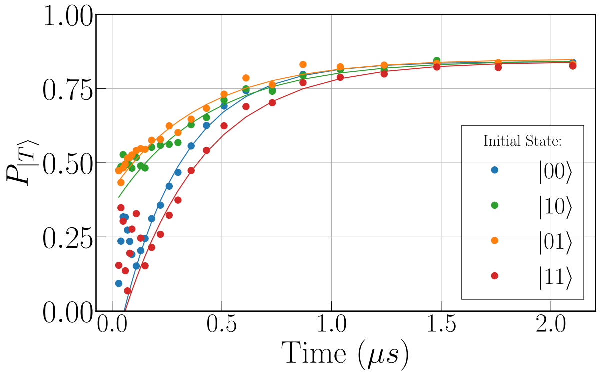


FIG. 7. Stabilization trajectory of the qutrit-qubit system into the Bell state $|T\rangle$ with the two transmons initialized into each of four states: $|00\rangle, |10\rangle, |01\rangle$ and $|11\rangle$. Measured data is presented as dots, with fits as solid lines. This demonstrates the universality of this stabilization protocol for states in the qubit-qubit space.

## SUPPLEMENTARY NOTE 5: EXPERIMENTAL IMPERFECTIONS AND CROSSTALK

### Robustness to parameter deviations

We simulated the sensitivity of our stabilization protocol to parameter imperfections and show the results in Supplementary Fig. 8. The protocol is relatively robust to drive detuning, so that a detuning of up to 1 MHz causes

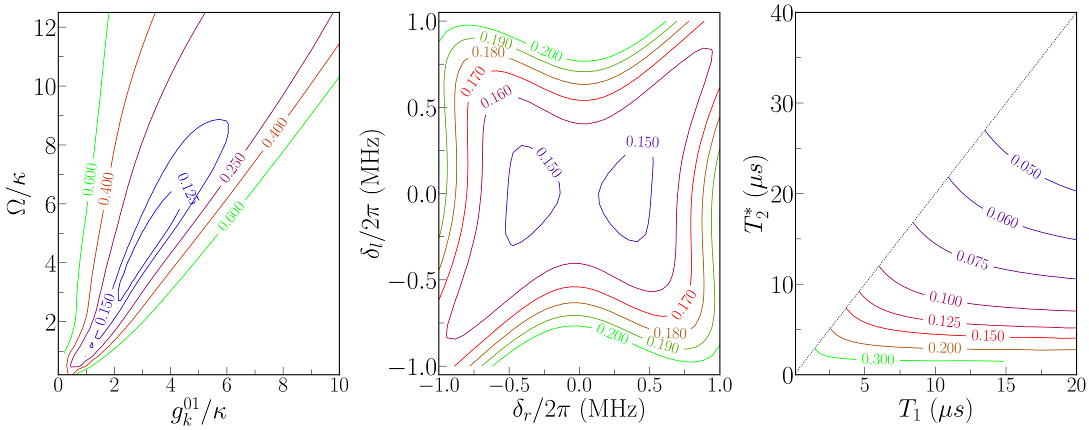


FIG. 8. Steady-state error as a function of (a) 0-1 direct drive amplitude and 0-1 sideband drive amplitude, both normalized to resonator linewidth $\kappa$, (b) detunings on the 0-1 transitions for left and right transmons, and (c) transverse and longitudinal relaxation times (assuming same decoherence rates for both transmons). The other parameters were fixed at the nominal values reported in Supplementary Table I

a drop in target state fidelity of only 4%. By improving the qubit coherence up to $T_1 = T_2^* \approx 20\mu s$, fidelities above 95% are achievable in the current scheme.

### Impact of leakage to higher transmon levels

In the main text we characterized our system by performing two-qubit quantum state tomography. This approach can be considered valid if we have negligible leakage out of the computational space into higher transmon levels. In particular leakage into the $|20\rangle$ and $|21\rangle$ energy levels is expected to be the dominant mechanism, because of the presence of the sideband drive targeting the 1-2 transition of qubit $l$. In order to determine the impact of such leakage, we measured the population of the second excited state $|2\rangle_l$ during stabilization by following the procedure in [10]. We can then use this information to constrain the trace of the tomographic reconstruction and estimate error bounds caused by leakage. Note that the latter measurement does not contain information about the $|20\rangle$ and $|21\rangle$ level populations independently, which would require a full 3-level tomographic reconstruction. We investigate the possible impact of leakage as well as potential unknown correlations in the qubit-qubitrit Hilbert by use of a Monte Carlo approach to examine the distribution of the log-negativity over the space of $6 \times 6$ qubit-qutrit density matrices that are compatible with the reconstructed qubit-qubit density matrix and measured leakage to $|2\rangle_l$, as explained below.

In the Monte Carlo study we first randomly choose the leakage population between the two levels, as shown in Supplementary Eq. (18) with random real variable $\zeta \in [0, 1]$,

$$\tilde{\rho} = \rho_{qq} + (1 - \mathrm{Tr}(\rho))((1-\zeta)|20\rangle\langle 20| + \zeta|21\rangle\langle 21|), \tag{18}$$

where $\rho_{qq}$ is the reconstructed density matrix from two-qubit state tomography. Supplementary Eq. (18) assumes that there are no residual correlations outside the qubit manifold, which we expect to be true to a good approximation as we approach the target state. Possible remaining correlations between the population in the qutrit $|2\rangle$ level may have an impact on the estimate of the state entanglement. To this end, we modify the density matrix in Supplementary Eq. (18) by adding correlations between the qubit and qutrit sectors, weighted with a randomized complex variable $C_{(\Phi,\Psi)}$ with $|C_{(\Phi,\Psi)}| \in [0, 1)$ and $\arg(C_{(\Phi,\Psi)}) \in [0, 2\pi)$,

$$\tilde{\rho}' = \tilde{\rho} + \sum_{\substack{\Phi \in S_{qq},\\ \Psi \in S_t}} \sqrt{\Phi^\dagger \tilde{\rho} \Phi \times \Psi^\dagger \tilde{\rho} \Psi}\left(C_{(\Phi,\Psi)} \Phi \Psi^\dagger + C^\dagger_{(\Phi,\Psi)} \Psi \Phi^\dagger\right) \tag{19}$$

where $S_{qq} = \{|00\rangle, |01\rangle, |10\rangle, |11\rangle\}$ and $S_t = \{|20\rangle, |21\rangle\}$. The validity of the resulting density matrix is checked by verifying that it is positive semi-definite within a margin of error of $1 \times 10^{-3}$. We apply this Monte Carlo analysis to the stabilized state density matrix measured at $t = 50\,\mu\text{s}$ [shown in the main text] using the method described above and compute the resulting logarithmic negativity and purity. The results of this analysis are shown in Supplementary Fig. 9. Our analysis confirms that the qutrit-qubit entanglement is extremely weak as most of the weight of the $6 \times 6$ density operators clusters near the value of logarithmic negativity of 77% consistent with the values reported in the main text based on the $4 \times 4$ qubit-qubit reconstruction. The purity is also consistent with the measured value, with

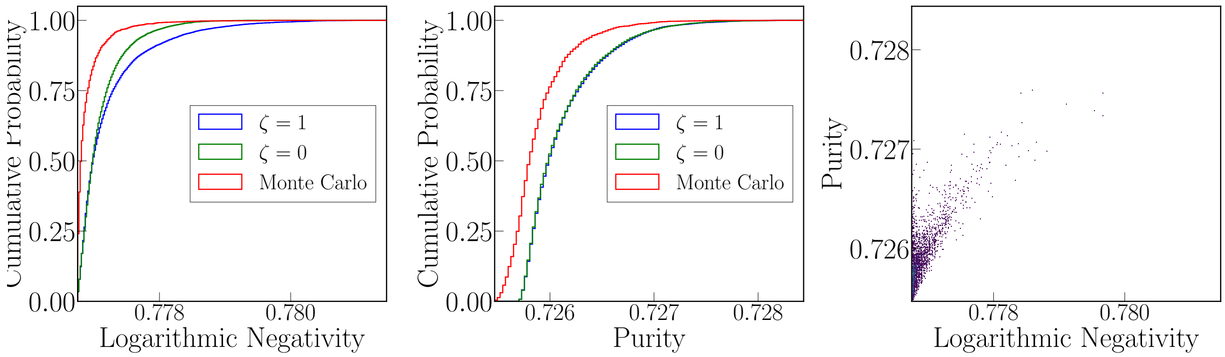


FIG. 9. Results of the Monte-Carlo entanglement bound study. Left, Center: logarithmic negativity and purity respectively for cases where the higher level population balance parameter $\zeta$ is randomized $\zeta$ case, as well as for the cases where $\zeta = 0$ and $\zeta = 1$. Right: histogram of the purity and logarithmic negativity based on Monte Carlo extension of the density operator.

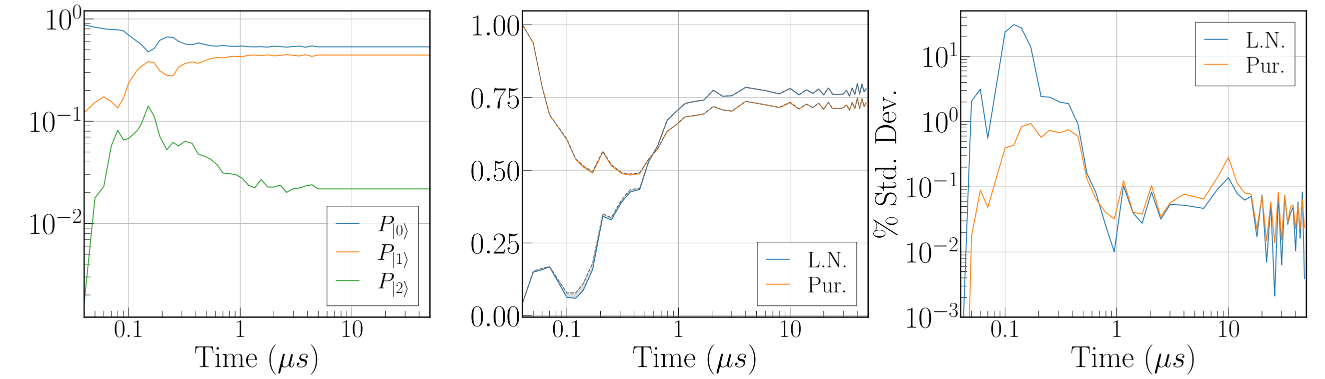


FIG. 10. Left: measured populations in the three levels of transmon $l$ over time. Center: results of the purity and logarithmic negativity from the Monte Carlo analysis: solid lines show the minimum value of the distribution, corresponding to the absence of correlation between the upper and lower levels and the most likely outcome at long times. Dashed lines are one standard deviation away from this case. Right: percentage standard deviation ($100\% \times \sigma/\text{Value}$) as a function of time, showing that the largest error caused by correlations occurs when the population in the upper level is at its peak.

any difference entirely accounted for by the population of the $|2\rangle_l$ state due to leakage (leading to a concomitant reduction in purity of $\rho_{qq}$). Furthermore, the maximum bound of entanglement is within 1% of the reported value as shown by the span of logarithmic negativity for the entire distribution of constructed density operators.

By applying this analysis to each data point allows us to estimate the potential impact of population in the higher levels on metrics of our prepared state over time for the full qutrit-qubit space. These calculations were done with 50k random samples in a Monte-Carlo configuration. The population in $|2\rangle$ peaks around 200 ns at $\approx$ 10%, then drops rapidly to its terminal value of $\approx$ 2% (see Supplementary Fig. 11, left). We then extracted the state purity and logarithmic negativity (Supplementary Fig. 11, middle). The minimum values of these distributions, corresponding to the case with no correlations between the qubit and qutrit subspaces, can be used as a lower bound on the purity and logarithmic negativity. We also computed the standard deviation (Supplementary Fig. 11, right) of the purity and logarithmic negativity distributions, which peaks at around 10% when the leakage population is maximum and then drops to $< 0.1\%$.

## Effect of crosstalk on error-time scaling

In Supplementary Fig. 11 we plot the measured product between preparation error and time $\varepsilon_\infty \tau$ from Fig. 4(b) in the main text versus the Rabi rate $\Omega/\Omega_{\text{opt}}$ normalized to the optimal Rabi rate. Note that the error-time product is non-monotonic and achieves its minimum value at the optimal Rabi rate $\Omega_{\text{opt}}$.

In order to quantitatively understand the error-time scaling observed in the experiment and diagnose the cause

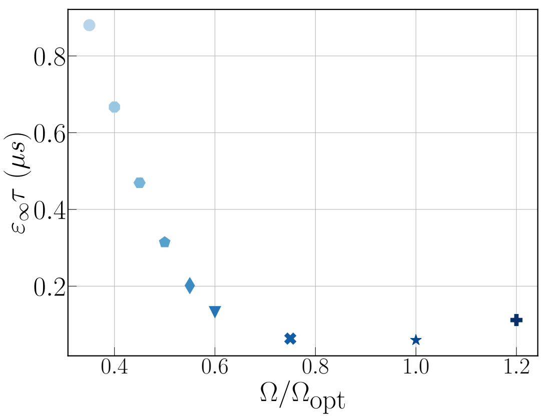


FIG. 11. Measured $\varepsilon_\infty \tau$ product plotted as a function of $\Omega/\Omega_{\text{opt}}$.

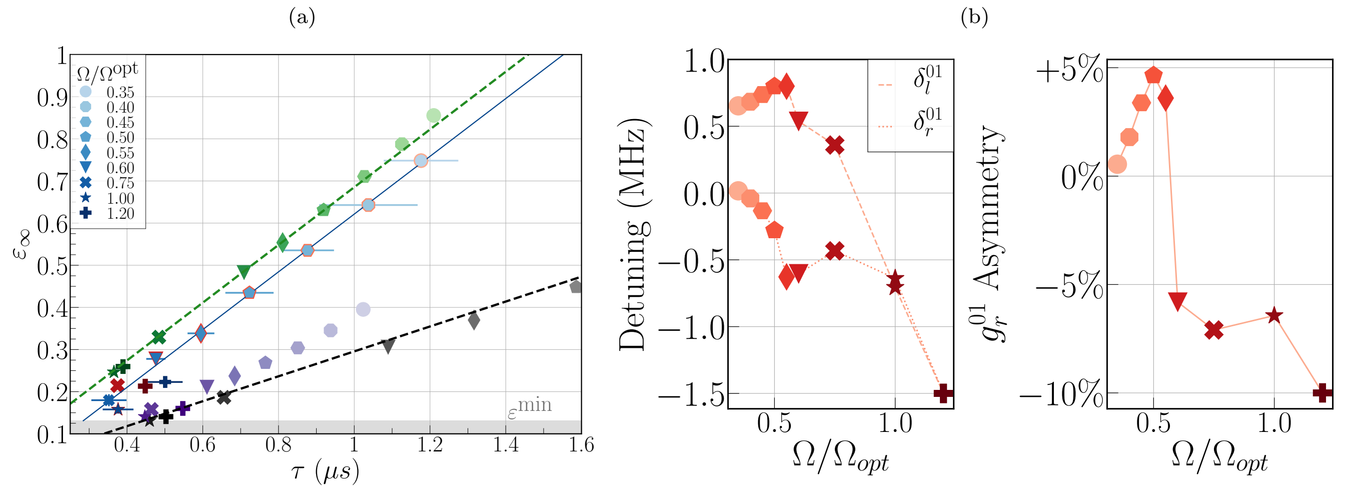


FIG. 12. (a) Comparison of data shown in Supplementary Fig. 4(b) of the main text, with simulations of the full qutrit-qubit and resonator system. Black markers correspond to master equation simulation shown in the Methods, using the experimental parameters listed in Supplementary Table I. The green markers are result of a similar simulation done with reduced coherence times to match the steeper slope of the data, assuming identical relaxation and transverse relaxation times for both transmons and $T_1 = T_2$ that yields slope as $3\gamma_{l,r}^{01}/2$. The purple markers are a result of simulation including a fixed detuning on each qubit, $\delta_l^{01} = 483$ kHz and $\delta_r^{01} = -342$ kHz. The red markers are a result of a simulation that optimizes the detunings and a small asymmetry in $g_{l,r}^{01}$ for each point to best fit the experimentally measured error-time (blue markers). In each case, $\Omega^{\rm opt}$ is set by the drive amplitude that realizes the minimum steady state error achievable in the given simulation model. (b) Values of $\delta_{l,r}^{01}$ and percentage asymmetry in $g_r^{01}$, extracted from fitting the experimentally measured steady state error and stabilization time as a function of normalized Rabi drive strength (red markers on left plot).

of deviations from the theoretically predicted performance, we perform detailed numerical studies summarized in Supplementary Fig. 12. The steady-state error and time estimated from full simulations of the master equation reported in Methods fall on a straight line with slope $1/T_B$ (dashed-black), confirming the prediction from the semi-classical rate equation model. Experimentally measured scaling of error-time, while linear, corresponds to a steeper slope though (solid-blue). A naive expectation of reduced transmon coherence could explain the steeper slope (dashed-green), but it is inconsistent with the magnitude of the minimum reported error $\varepsilon_\infty^{\rm min}$. The next potential cause of this discrepancy could be a drive-induced Stark shift or detuning which leads to coherent leakage out of the target state; as shown by the simulations, assuming a constant detuning leads to a qualitatively different variation between $\varepsilon_\infty$ and $\tau$. On the other hand, accounting for drive-dependent crosstalk both as a Stark shift on 0-1 frequencies and an imbalance in 0-1 sideband amplitudes can simultaneously describe the faster slope and minimum error observed in the experiment. We therefore conclude that this is the most likely explanation for the increased slope.

## SUPPLEMENTARY NOTE 6: E-BIT GENERATION CAPACITY

To obtain the e-bit generation capacity reported in Table 4(c) of the main text, we assume the input to stabilization channel as a sequence of bit-pairs, where number of bit pairs processed by the channel, $n_c = T_c/\tau$, is set by the repetition time of the experiment $T_c$ and stabilization time constant $\tau$. Modeling each run as a binomial process, with a success probability, $p(t) = 1 - \varepsilon(t)$, and failure probability, $q(t) = 1 - p(t) = \varepsilon(t)$, one can use the central limit theorem in the limit of large number of repetitions to obtain the "efficiency" of such a continuous channel that generates e-bits at a fixed bandwidth (set by $W \equiv \tau/T_c$) and constant noise (set by the error $\varepsilon(T_c)$). Using the Shannon's formula for efficiency of fixed-bandwidth channels, along with the expressions for maximum output entropy $H_{\rm max} = \log_2(2\pi e(S_e + N_e))$ and conditional entropy $H_{\rm error} = \log_2(2\pi e N_e)$, where $S_e = n_c^2(1-\varepsilon(t))^2$ and $N_e = n_c\varepsilon(t)(1-\varepsilon(t))$ are the root-mean-square signal and noise of the distribution, one obtains

$$\begin{aligned}\mathcal{E}_e &\equiv W \log_2\left(1 + \frac{1}{W}\frac{S_e}{N_e}\right), \\ &= \frac{\tau}{T_C}\log_2\left(1 + \frac{1-\varepsilon(t)}{\varepsilon(t)}\frac{T_c}{\tau}\right). \end{aligned} \tag{20}$$

From efficiency $\mathcal{E}_e$, we can straightforwardly write the information (here number of e-bits) generation capacity as, $I_e(T_c) \equiv (T_C/\tau)\mathcal{E}_e$. It is instructive to note the two limiting cases:

- $T_C \ll \tau$ or *bandwidth-limited regime*: In this regime, $I_e \approx C \times (T_c/\tau)^2$ with $C = (F_\infty^{\max}/\varepsilon_\infty^{\min})\log_2 e$, i.e. faster the scheme (or smaller the $\tau$) higher the e-bit generation capacity of the protocol.

- $T_C \gg \tau$ or *noise-limited regime*: In this regime, the capacity grows logarithmically being almost entirely limited by noise (steady state error here); this is consistent with the fact that when the channel is used slowly enough, the capacity is set by steady-state performance metrics.

The different scaling of the generation capacity in the two regimes is evident from the plots shown in Supplementary Fig. 13.

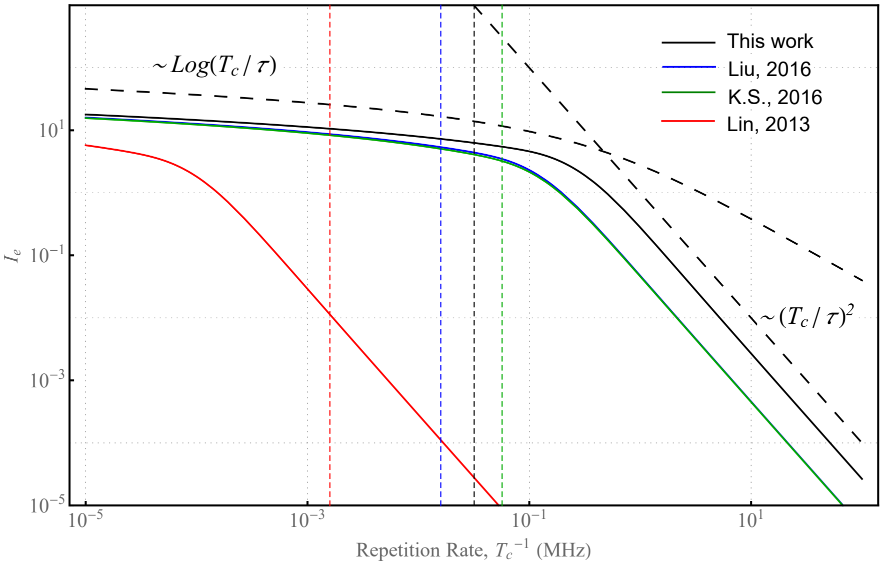


FIG. 13. Plots of e-bit generation capacity, $I_e(T_c)$, for the four experiments reported in Table 4(c) of the main text (including this work) [17–19]. The vertical dashed lines indicate the dephasing rate corresponding to $T_2^*$ for each experiment.


[1] E. Zakka-Bajjani, F. Nguyen, M. Lee, L. R. Vale, R. W. Simmonds, and J. Aumentado, Nature Physics **7**, 599 (2011).
[2] A. J. Sirois, M. Castellanos-Beltran, M. DeFeo, L. Ranzani, F. Lecocq, R. Simmonds, J. Teufel, and J. Aumentado, Applied Physics Letters **106**, 172603 (2015).
[3] B. Kraus, H. P. Büchler, S. Diehl, A. Kantian, A. Micheli, and P. Zoller, Physical Review A **78**, 042307 (2008).
[4] K. Stannigel, P. Rabl, and P. Zoller, New Journal of Physics **14**, 063014 (2012), ISSN 1367-2630.
[5] F. Motzoi, E. Halperin, X. Wang, K. B. Whaley, and S. Schirmer, Physical Review A **94**, 032313 (2016).
[6] E. Doucet, F. Reiter, L. Ranzani, and A. Kamal, Phys. Rev. Research **2**, 023370 (2020).
[7] Z. Leghtas, U. Vool, S. Shankar, M. Hatridge, S. M. Girvin, M. H. Devoret, and M. Mirrahimi, Phys. Rev. A **88**, 023849 (2013).
[8] C. A. Ryan, B. R. Johnson, D. Ristè, B. Donovan, and T. A. Ohki, Review of Scientific Instruments **88**, 104703 (2017).
[9] J. Koch, M. Y. Terri, J. Gambetta, A. A. Houck, D. I. Schuster, J. Majer, A. Blais, M. H. Devoret, S. M. Girvin, and R. J. Schoelkopf, Physical Review A **76**, 042319 (2007).
[10] M. J. Peterer, S. J. Bader, X. Jin, F. Yan, A. Kamal, T. J. Gudmundsen, P. J. Leek, T. P. Orlando, W. D. Oliver, and S. Gustavsson, Phys. Rev. Lett. **114**, 010501 (2015).
[11] J. Gambetta, A. Blais, D. I. Schuster, A. Wallraff, L. Frunzio, J. Majer, M. H. Devoret, S. M. Girvin, and R. J. Schoelkopf, Phys. Rev. A **74**, 042318 (2006).
[12] J. Li, G. Paraoanu, K. Cicak, F. Altomare, J. I. Park, R. W. Simmonds, M. A. Sillanpää, and P. J. Hakonen, Physical Review B **84**, 104527 (2011).
[13] Y. Lu, S. Chakram, N. Leung, N. Earnest, R. K. Naik, Z. Huang, P. Groszkowski, E. Kapit, J. Koch, and D. I. Schuster, Physical review letters **119**, 150502 (2017).
[14] C. A. Ryan, B. R. Johnson, J. M. Gambetta, J. M. Chow, M. P. da Silva, O. E. Dial, and T. A. Ohki, Physical Review A **91**, 022118 (2015).

[15] S. Filipp, P. Maurer, P. J. Leek, M. Baur, R. Bianchetti, J. Fink, M. Göppl, L. Steffen, J. M. Gambetta, A. Blais, et al., Physical review letters **102**, 200402 (2009).
[16] M. Steffen, M. Ansmann, R. C. Bialczak, N. Katz, E. Lucero, R. McDermott, M. Neeley, E. M. Weig, A. N. Cleland, and J. M. Martinis, Science **313**, 1423 (2006).
[17] Y. Liu, S. Shankar, N. Ofek, M. Hatridge, A. Narla, K. Sliwa, L. Frunzio, R. J. Schoelkopf, and M. H. Devoret, Physical Review X **6**, 011022 (2016).
[18] M. Kimchi-Schwartz, L. Martin, E. Flurin, C. Aron, M. Kulkarni, H. Tureci, and I. Siddiqi, Physical Review Letters **116**, 240503 (2016).
[19] Y. Lin, J. Gaebler, F. Reiter, T. R. Tan, R. Bowler, A. Sørensen, D. Leibfried, and D. J. Wineland, Nature **504**, 415 (2013).
